# Induction of antiviral interferon-stimulated genes by neuronal STING promotes the resolution of pain in mice

**DOI:** 10.1172/JCI176474

**Published:** 2024-03-19

**Authors:** Manon Defaye, Amyaouch Bradaia, Nasser S. Abdullah, Francina Agosti, Mircea Iftinca, Mélissa Delanne-Cuménal, Vanessa Soubeyre, Kristofer Svendsen, Gurveer Gill, Aye Ozmaeian, Nadine Gheziel, Jérémy Martin, Gaetan Poulen, Nicolas Lonjon, Florence Vachiery-Lahaye, Luc Bauchet, Lilian Basso, Emmanuel Bourinet, Isaac M. Chiu, Christophe Altier

**Affiliations:** 1 Department of Physiology and Pharmacology, Cumming School of Medicine,; 2 Inflammation Research Network–Snyder Institute for Chronic Diseases, Cumming School of Medicine, and; 3 Alberta Children’s Hospital Research Institute, University of Calgary, Calgary, Alberta, Canada.; 4 Department of Neurosurgery, Gui de Chauliac Hospital, Donation and Transplantation Coordination Unit, Montpellier University Medical Center, Montpellier, France.; 5 Hotchkiss Brain Institute, Cumming School of Medicine, University of Calgary, Calgary, Alberta, Canada.; 6 Toulouse Institute for Infectious and Inflammatory Diseases (INFINITy), INSERM UMR1291, University of Toulouse III, Toulouse, France.; 7 Institute of Functional Genomics, Montpellier University, CNRS, INSERM, Montpellier, France.; 8 Department of Immunology, Blavatnik Institute, Harvard Medical School, Boston, Massachusetts, USA.

**Keywords:** Inflammation, Neuroscience, Innate immunity, Ion channels, Pain

## Abstract

Inflammation and pain are intertwined responses to injury, infection, or chronic diseases. While acute inflammation is essential in determining pain resolution and opioid analgesia, maladaptive processes occurring during resolution can lead to the transition to chronic pain. Here we found that inflammation activates the cytosolic DNA–sensing protein stimulator of IFN genes (STING) in dorsal root ganglion nociceptors. Neuronal activation of STING promotes signaling through TANK-binding kinase 1 (TBK1) and triggers an IFN-β response that mediates pain resolution. Notably, we found that mice expressing a nociceptor-specific gain-of-function mutation in STING exhibited an IFN gene signature that reduced nociceptor excitability and inflammatory hyperalgesia through a KChIP1-Kv4.3 regulation. Our findings reveal a role of IFN-regulated genes and KChIP1 downstream of STING in the resolution of inflammatory pain.

## Abstract 2

STING is a marker of pain sensitization, and interferon-stimulated genes serve as key regulators of nociceptive ion channels and ion channel&ndash;associated proteins during pain resolution.

## Graphical abstract

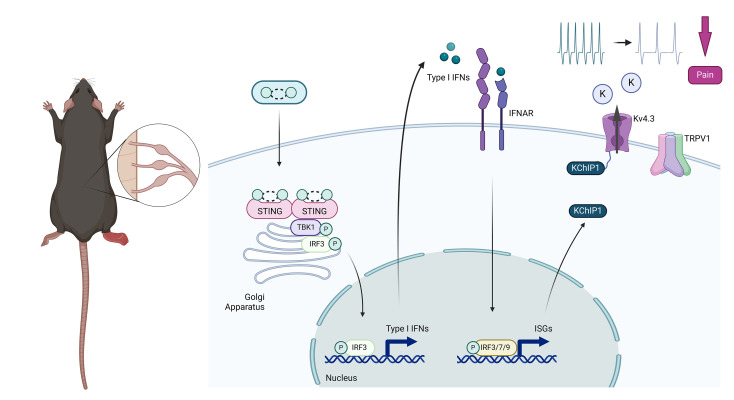

## Introduction

Pain and inflammation are two integrated biological responses that serve as the main defense mechanisms during injury or infection. Inflammatory pain is adaptive and protective; however, it often persists even after the inflammation has subsided. While it is well established that activated immune cells contribute to acute pain (1, 2), recent studies suggest that acute inflammation can also be protective against the transition to chronic pain (3). Therefore, understanding how pain resolves during inflammation is crucial for preventing the development of chronic pain.

Like the immune system, the nociceptive system alerts the host to the presence of “danger signals” (4), and certain pathogens have evolved mechanisms to disable sensory modalities, such as smell, taste (5–7), and even pain (6), by targeting danger signal receptors. Nociceptors have the ability to respond to pathogen- and damage-associated molecular patterns through various pattern recognition receptors (8). TLRs, NOD-like receptors (NLRs), RIG-I–like receptors (RLRs), and cytosolic DNA sensors (CDSs) have been reported to be expressed in dorsal root ganglion (DGR) neurons (8).

Among these receptors, stimulator of IFN genes (STING) is a cytosolic DNA sensor that plays a role in recognizing self-DNA, viral DNA, and cyclic dinucleotides (CDNs; c-di-GMP, 3′3′-cGAMP, and c-di-AMP) produced by bacteria. STING activation leads to the production of type I IFNs (IFN-I) such as IFN-α and IFN-β isoforms. Bacterial and host-derived CDNs induce STING dimerization, which activates TANK-binding kinase 1 (TBK1). TBK1 phosphorylates IFN regulatory factor 3 (IRF3), leading to the production of IFN-I and other genes involved in host responses, promoting elimination of pathogens and damaged host cells upon inflammation (9). Type I IFNs bind to IFN-α/β receptor (IFNAR) on producer and nearby cells. This autocrine and paracrine stimulation leads to the transcriptional regulation of a wide array of IFN-stimulated genes (ISGs) that ordinarily protect cells from infection. Although IFNs were discovered owing to their antiviral properties, they are currently approved for treating a variety of diseases including hepatitis, multiple sclerosis, and melanoma (10).

Recently, STING has emerged as a regulator of nociception (11–15). Mice lacking STING developed mechanical allodynia, and STING agonists produce anti-nociceptive effects in neuropathic pain models (11). Despite the observed analgesic properties of STING agonists, the role of STING in pathological pain and the mechanisms by which STING modulates and reprograms nociceptors in inflammatory pain remain unclear. Moreover, the STING/IFN-I pathway is known to be activated during inflammation, and its dysregulation can contribute to chronic inflammatory conditions. Exploring the role of this pathway in the resolution of inflammatory pain can provide insight into preventing the transition to chronic pain in inflammatory conditions. Here, using transcriptional analysis of sensitized nociceptive neurons, we examined the putative genes that contribute to the phenotypic plasticity of nociceptors in inflammation-induced sensitization and its resolution. We found that inflammation induced by complete Freund’s adjuvant (CFA) leads to an upregulation of STING in Nav1.8^+^ and TRPV1^+^ neurons. We show that mice expressing a TRPV1 nociceptor–specific gain-of-function mutation in human STING exhibited an IFN gene signature associated with reduced heat hyperalgesia. Notably, we identified several IFN-regulated genes (IRGs) as nociceptor-specific ion channels, including TRPV1 and KChIP1, which control nociceptor excitability and hyperalgesia. Overall, our findings suggest that STING serves as a marker of nociceptor sensitization, and IRGs play a crucial role in regulating persistent pain conditions. This work provides novel mechanistic insights into the role of the STING/IFN-I pathway in the regulation of key ion channels and ion channel–associated proteins for the resolution of inflammatory pain.

## Results

### Nociceptor STING is upregulated in response to inflammation.

**Figure 1:**
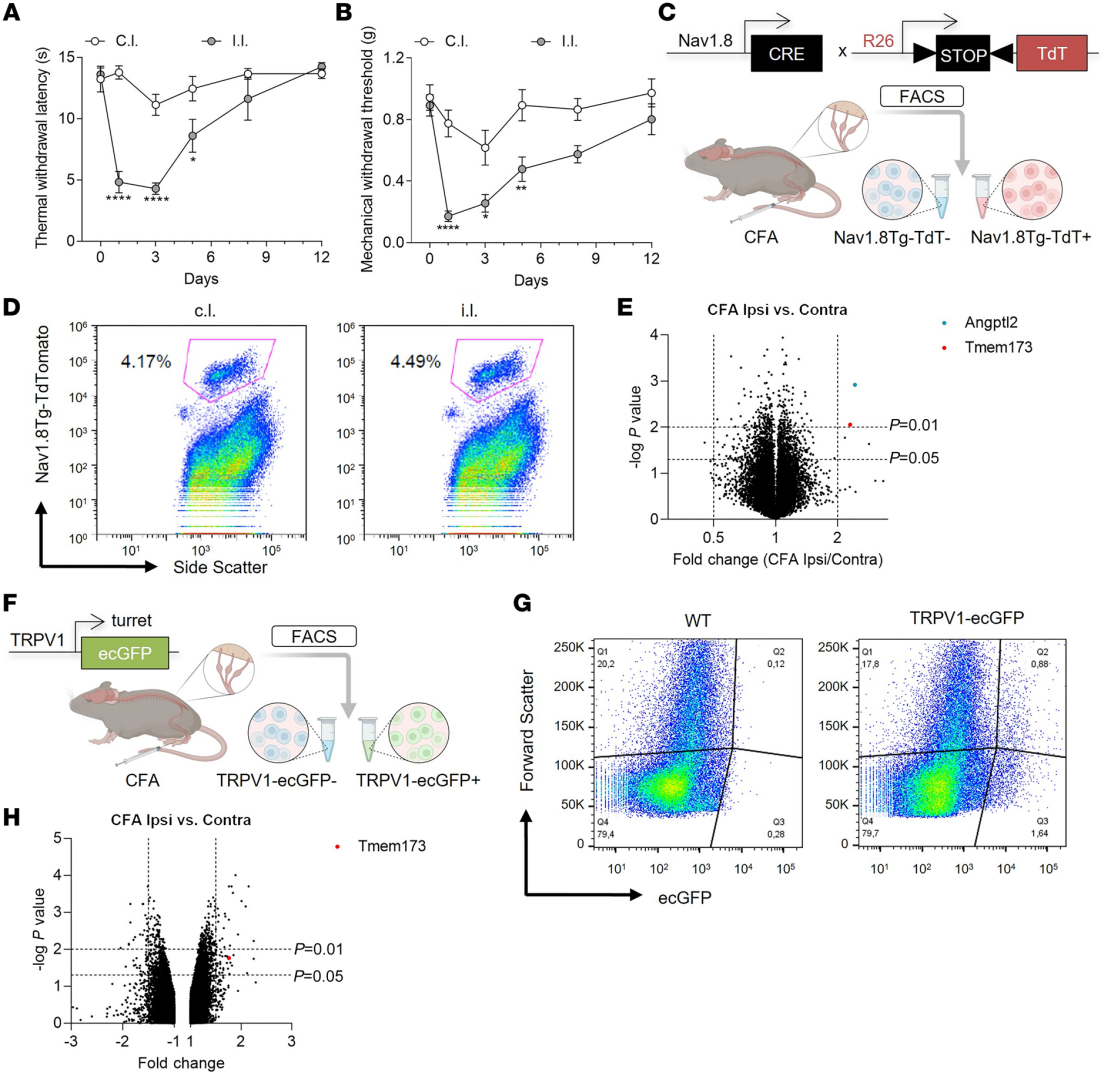
Transcriptional changes in Nav1.8 and TRPV1 neurons after CFA inflammation. (**A**) Measurement of thermal withdrawal latency in the ipsilateral (i.l.) and contralateral (c.l.) hind paws of C57BL/6J mice treated with CFA (*n* = 6). (**B**) Measurement of mechanical withdrawal threshold in the ipsilateral and contralateral hind paws of C57BL/6J mice treated with CFA (*n* = 8). (**C**) Experimental approach used to isolate *Nav1.8 Tg-TdTomato* neurons for microarray analysis, 24 hours after intraplantar CFA. (**D**) FACS plots are shown as representative example of the gating strategy used for *Nav1.8-Cre Tg-TdTomato* lumbar DRG neuron isolation from contralateral and ipsilateral sides following CFA injection. Lumbar DRGs were pooled from 3 mice per sample. (**E**) Volcano plot showing transcriptional changes induced by CFA inflammation in *Nav1.8 Tg-TdT* neurons (*n* = 3 mice). *P* value line cutoff is *P* < 0.01, and fold change of 2. Select transcripts of interest are highlighted in distinct colors (inset legend). (**F**) Experimental approach used to isolate *TRPV1-pHluorin* neurons for microarray analysis, 72 hours after intraplantar CFA. (**G**) FACS plots are shown as representative example of the gating strategy used for WT and *TRPV1-pHluorin* DRG neuron isolation from ipsilateral side following CFA injection. Lumbar DRGs were pooled from 3 mice per sample. (**H**) Volcano plot showing transcriptional changes induced by CFA inflammation in TRPV1 neurons. *P* value line cutoff is *P* < 0.05, and fold change of 1.5. Select transcript of interest is highlighted in red (inset legend). Statistical analysis was performed using 2-way ANOVA followed by Šidák’s post hoc test (**A** and **B**; *P < 0.05, **P < 0.01, ****P < 0.0001).

To study the mechanisms of resolution of inflammatory pain, we used the well-established inflammatory pain model of complete Freund’s adjuvant (CFA), which induces local inflammatory responses to the injection of heat-killed *Mycobacterium tuberculosis*. Mice developed thermal hyperalgesia (Figure 1A) and mechanical allodynia (Figure 1B) by day 1 after CFA injection and recovered within 2 weeks, suggesting a high level of plasticity in the afferent pain pathway, particularly in unmyelinated small-diameter nociceptors that undergo significant molecular and functional changes during inflammation (16, 17). To explore how nociceptors respond to the inflammatory milieu, we used *Nav1.8 Tg-TdTomato* mice, which mark neurons that express the voltage-gated sodium channel Nav1.8 (*Scn10a*) (18). After intraplantar CFA injection, we FACS-sorted Nav1.8^+^ neurons from paw-innervating ipsilateral and contralateral lumbar (L4–L6) DRGs (Figure 1, C and D) (19). Transcriptional analysis identified 2 differentially expressed genes between ipsilateral and contralateral sides: *STING* (*Tmem173* gene) and angiopoietin-like protein 2 (*Angptl2*) (Figure 1E and Supplemental Table 1; supplemental material available online with this article; https://doi.org/10.1172/JCI176474DS1). To narrow down our analysis to TRPV1^+^ nociceptors that play a central role in inflammatory hyperalgesia (20), we repeated the experiment using a *TRPV1-pHluorin* mouse previously characterized (Figure 1, F and G) (21). As observed with the larger population of Nav1.8^+^ neurons, we found that CFA inflammation enhanced *STING* expression in ipsilateral TRPV1^+^ neurons (Figure 1H). Co-immunostaining of STING and TRPV1-ecGFP in lumbar ganglia from CFA-treated animals (Supplemental Figure 1A) validated the transcriptomic data and showed an increase in STING protein level in ipsilateral TRPV1^+^ neurons (Supplemental Figure 1B). Analyzing a single-cell RNA-Seq data set of mouse DRG neurons (22), we found that STING was coexpressed with peptidergic nociceptor-associated transcripts such as *Scn10a* (Nav1.8), *Trpv1*, *Calca* (CGRP), *Tac1* (substance P), and *GFRα3* (Supplemental Figure 2A). We thus performed immunostaining to determine the level of STING protein in the different subpopulations of DRG neurons: TRPV1 (PEP1.1, PEP1.2, PEP1.3, PEP1.4, NP3) and IB4 (NP1, NP2, NP3, TH) (Supplemental Figure 2B). Immunostaining analysis confirmed that STING was enriched in TRPV1^+^ peptidergic neurons (PEP1.1, PEP1.2, PEP1.3, PEP1.4, NP3) compared with IB4^+^ non-peptidergic neurons (NP1, NP2, NP3, TH) (Supplemental Figure 2C). Furthermore, we evaluated the expression of *STING* in human DRGs (Supplemental Figure 2D). We found a high degree of colocalization between *STING* and *Nav1.8* transcripts, confirming that STING was enriched in neuronal rather than non-neuronal cells of human DRGs (Supplemental Figure 2E). Collectively, our results confirmed that STING is expressed in both human and mouse nociceptors.

### Type I IFNs promote resolution of inflammatory pain.

**Figure 2:**
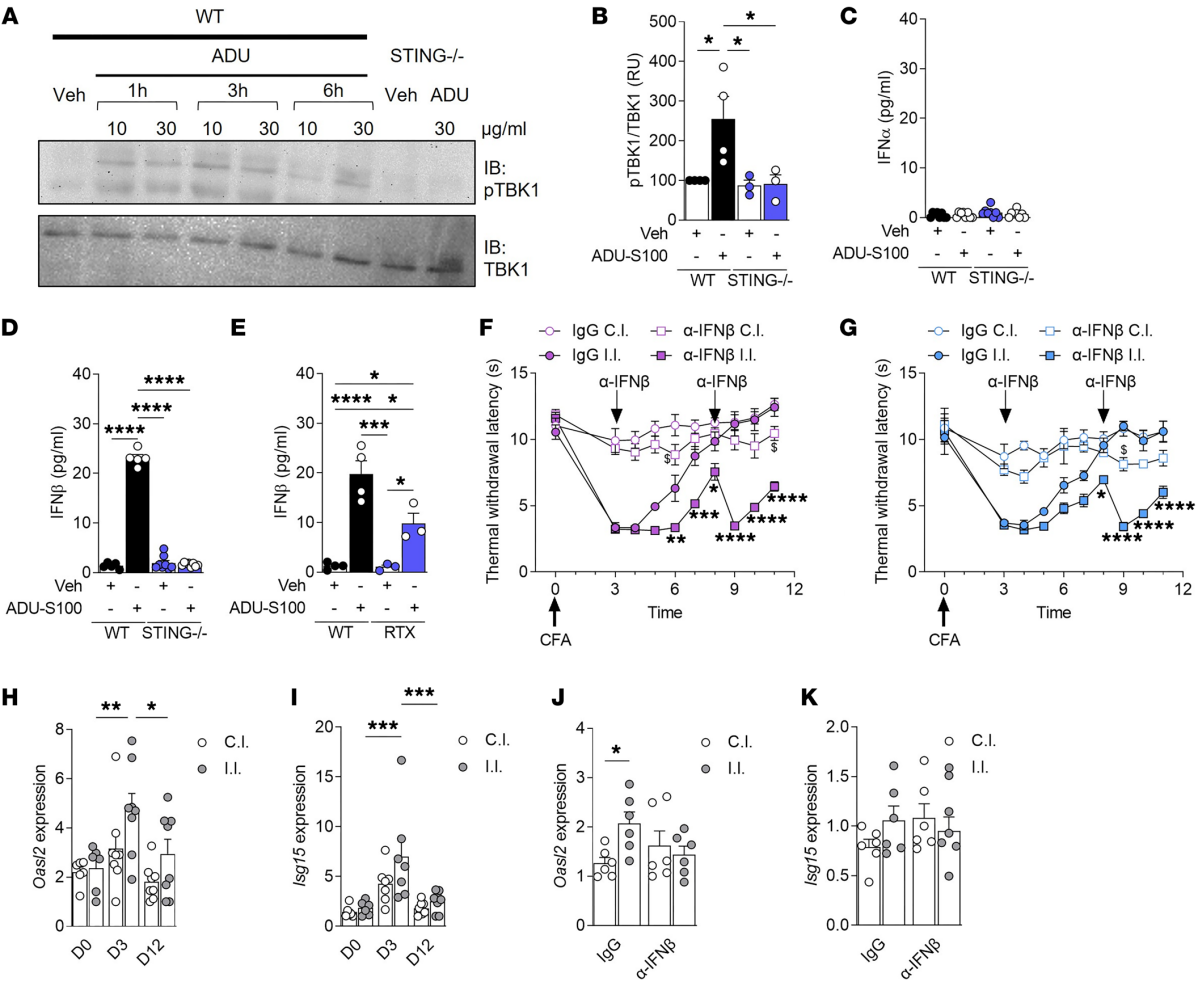
Neuronal type I IFNs promote resolution of inflammatory pain. (**A**) Phospho-TBK1 protein level was determined by Western blot in lumbar DRG culture (L4–L6) treated with vehicle or ADU-S100 (10 or 30 μg/mL) for 1, 3, or 6 hours, from WT (*n* = 4) and *STING^–/–^* (*n* = 3) mice. Three independent experiments were performed. (**B**) Phospho-TBK1 quantification at 3 hours in response to 10 μg/mL of ADU-S100. Data are normalized to TBK1 signal. (**C** and **D**) IFN-α (**C**) and IFN-β (**D**) levels were determined in the DRG culture of WT (*n* = 5–8) and *STING^–/–^* (*n* = 8) mice treated with vehicle or ADU-S100 (10 μg/mL). (**E**) IFN-β levels in vehicle-pretreated (*n* = 4) and RTX-pretreated (*n* = 3) DRG culture, stimulated with ADU-S100. Two vehicle samples from WT mice were used for both **D** and **E**, as these conditions were run simultaneously. (**F** and **G**) Measurement of thermal withdrawal latency in hind paws of female (**F**) or male (**G**) CFA-treated C57BL/6J mice that received either an IgG control (*n* = 5–6) or an IFN-β neutralizing antibody at days 3 and 8 after CFA injection (*n* = 5–6). (**H** and **I**) *Oasl2* (**H**) and *Isg15* (**I**) mRNA expression in ipsilateral and contralateral lumbar DRGs of naive C57BL/6J mice (D0, *n* = 6) and mice 3 days (D3, *n* = 7–8) and 12 days after CFA injection (D12, *n* = 8). (**J** and **K**) *Oasl2* (**J**) and *Isg15* (**K**) expression at day 9 of CFA-treated mice that received either an IgG control (*n* = 6) or an IFN-β neutralizing antibody (*n* = 6). Statistical analysis was performed using 1-way ANOVA followed by Tukey’s post hoc test (**B**, **D**, and **E**; *P < 0.05, ***P < 0.001, ****P < 0.0001), Kruskal-Wallis followed by Dunn’s post hoc test (**C**), and 2-way ANOVA followed by Tukey’s post hoc test (**F** and **G**: *P < 0.05, **P < 0.01, ***P < 0.001, ****P < 0.0001 vs. IgG i.l.; ^$^*P* < 0.05 vs. IgG c.l.; **H** and **I**: *P < 0.05, **P < 0.01, ***P < 0.001) or by Bonferroni’s post hoc test (**J** and **K**; *P < 0.05).

STING activation in immune or epithelial cells leads to the production of type I IFNs (IFN-I) such as IFN-α and IFN-β isoforms. This IFN-I response is mediated by the recruitment of TBK1 and its phosphorylation (23). Accordingly, activation of STING by ADU-S100 (10 and 30 μg/mL) in cultured DRG neurons led to the phosphorylation of TBK1 in WT but not STING^–/–^ neurons (Figure 2, A and B). Additionally, ADU-S100 (10 μg/mL) stimulation induced the production of IFN-β, but not IFN-α, and this IFN-I response was dependent on the presence of STING (Figure 2, C and D). Thirteen distinct cell clusters have been identified in the DRG (24), including macrophages, fibroblasts, and satellite glial cells, which express STING (http://mousebrain.org/) (22). To assess the proportion of IFN-β produced by TRPV1-expressing neurons among all DRG cells, cultures were treated with resiniferatoxin (RTX) (1 μM) to ablate TRPV1^+^ neurons (Supplemental Figure 3, A and B) before ADU-S100 exposure. RTX pretreatment reduced the amount of IFN-β produced in response to the STING agonist by 50%, indicating that a substantial amount of IFN-I originated from TRPV1^+^ neurons (Figure 2E).

To investigate whether IFN-β mediated the resolution of thermal hyperalgesia, we used an anti–IFN-β neutralizing antibody. Mice subjected to CFA received intrathecal injections of anti–IFN-β antibody (30 ng/μL) or an IgG control (30 ng/μL) on the third and eighth days after CFA injection. Neutralizing anti–IFN-β delayed the resolution of thermal hyperalgesia in both female (Figure 2F) and male (Figure 2G) mice, suggesting a sex-independent role of IFN-I in pain resolution. Type I IFNs bind to IFN-α/β receptor (IFNAR) on producer and nearby cells. This autocrine and paracrine stimulation leads to the transcriptional regulation of a wide array of IFN-stimulated genes (ISGs) that ordinarily protect cells from infection. We thus assessed the expression of classical ISGs, specifically *Oasl2* and *Isg15*, over time in CFA-treated mice. Our findings revealed an increase of both *Oasl2* and *Isg15* three days after CFA injection in ipsilateral DRGs, returning to baseline levels by day 12 (Figure 2, H and I), suggesting that the resolution of CFA-induced hyperalgesia might be induced by a robust IFN signature. Accordingly, the expression of the *Oasl2* gene was reduced in hyperalgesic mice receiving anti–IFN-β, compared with normogesic mice injected with IgG control (Figure 2J). However, this observation was not found with the *Isg15* gene, which remained elevated in response to anti–IFN-β injection (Figure 2K), likely involving other STING-induced type I IFNs such as IFN-α.

### IFN-I/IFNAR1 signaling axis regulates thermal hyperalgesia.

**Figure 3:**
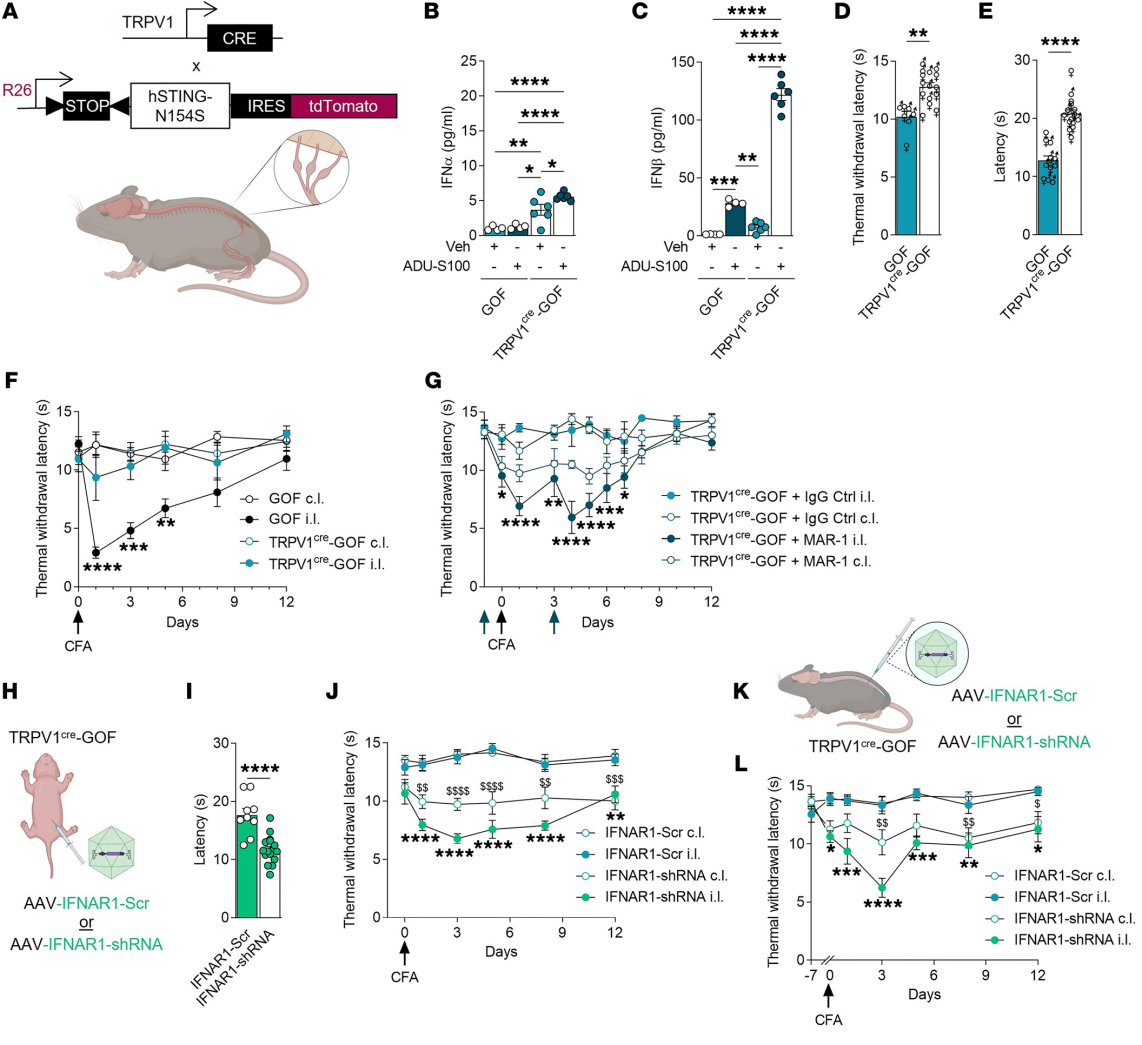
Nociceptor-specific *STING-N154S* gain of function reduces thermal sensitivity and heat hyperalgesia in an IFNAR1-dependent manner. (**A**) Schematic representation of transgenic *TRPV1^cre^-GOF* cKI mouse design. (**B** and **C**) IFN-α (**B**) and IFN-β (**C**) levels in DRG cultures of *GOF* (*n* = 4) and *TRPV1^cre^-GOF* (*n* = 6) mice stimulated with ADU-S100 (1 μg/mL). (**D** and **E**) Measurement of thermal sensitivity of naive *TRPV1^cre^-GOF* (*n* = 15–16) and *GOF* (*n* = 7–12) mice using the hot plate (**D**) or Hargreaves test (**E**). (**F**) Measurement of thermal withdrawal latency in hind paws of CFA-treated *GOF* (*n* = 9) and *TRPV1^cre^-GOF* (*n* = 8) mice. (**G**) Measurement of thermal withdrawal latency in hind paws of CFA-treated *TRPV1^cre^-GOF* mice that received either IgG control (*n* = 5) or IFNAR1 neutralizing antibody (MAR1) before and 3 days after CFA injection (*n* = 6). (**H**) Newly born *TRPV1^cre^-GOF* pups (P5) were given 10 μL of *AAV-PHP.S-DIO-IFNAR1-shRNA* or *AAV-PHP.S-DIO-scrambled-shRNA* intraperitoneally. (**I**) Measurement of thermal sensitivity of mice injected with *IFNAR1-Scr* (*n* = 9) or *IFNAR1-shRNA* (*n* = 16) AAV using the hot plate. (**J**) Measurement of thermal withdrawal latency of CFA-treated mice infected with *IFNAR1-Scr* (*n* = 7) or *IFNAR1-shRNA* (*n* = 9) AAV. (**K**) Adult *TRPV1^cre^-GOF* mice received 10 μL of *AAV-DIO-IFNAR1-shRNA* or *AAV-DIO-scrambled-shRNA* intrathecally. (**L**) Measurement of thermal withdrawal latency in hind paws of CFA-treated mice injected with *IFNAR1-Scr* (*n* = 7) or *IFNAR1-shRNA* (*n* = 8) AAV. Statistical analysis was performed using 1-way ANOVA followed by Tukey’s post hoc test (**B** and **C**; *P < 0.05, **P < 0.01, ***P < 0.001, ****P < 0.0001); *t* test (**E** and **I**) or Mann-Whitney test (**D**; **P < 0.01, ****P < 0.0001); and 2-way ANOVA followed by Tukey’s post hoc test (**F**: **P < 0.01, ***P < 0.001, ****P < 0.0001 vs. *TRPV1^cre^-GOF* i.l.; **G**: *P < 0.05, **P < 0.01, ***P < 0.001, ****P < 0.0001 vs. *TRPV1^cre^-GOF*+IgG i.l.; **J** and **L**: *P < 0.05, **P < 0.01, ***P < 0.001, ****P < 0.0001 vs. *IFNAR1-Scr* i.l.; ^$^*P* < 0.05, ^$$^*P* < 0.01, ^$$$^*P* < 0.001, ^$$$$^*P* < 0.0001 vs. *IFNAR1-Scr* c.l.).

To investigate the contribution of neuronal IFN-I in regulating nociceptive behaviors, we expressed a STING gain-of-function (GOF) mutant in TRPV1 neurons. The *hSTING* mutation (N154S) has been reported in STING-associated vasculopathy with onset in infancy (SAVI), a type I interferonopathy associated with constitutive activity of STING (25). We generated *TRPV1^cre^-GOF* conditional knockin (cKI) mice by crossing *hSTING-N154S* mice with *TRPV1-Cre* animals (Figure 3A). When assessing the efficiency of Cre recombination and the expression of *hSTING-N154S* in TRPV1^+^ neurons (Supplemental Figure 4A), we made 2 important observations: (a) the number of TRPV1^+^ neurons in the *TRPV1^cre^-GOF* was reduced in comparison with littermate controls (Supplemental Figure 4B), and (b) the large majority of TRPV1^+^ neurons expressed the *hSTING-N154S* mutation (Supplemental Figure 4C). We observed a similar trend with the peptidergic GFRα3 marker, while the expression of other neuronal markers, such as SP or GFRα2, remained similar in both *TRPV1^cre^-GOF* and littermate neurons (Supplemental Figure 5, A–F).

As TRPV1 nociceptors send their projection to the dorsal horn of the spinal cord, we examined the expression of TdTomato in the central terminals of nociceptors in laminae I and II (Supplemental Figure 6). Immunohistochemical analysis led to 2 observations: (a) *hSTING-N154S* expression occurs mainly in substance P (peptidergic) (Supplemental Figure 6A) and GFRα3 (peptidergic) nociceptors (Supplemental Figure 6B), with fewer IB4 (non-peptidergic) nociceptors (Supplemental Figure 6C); and (b) the neuroanatomical organization of peptidergic and non-peptidergic fibers was conserved in *TRPV1^cre^-GOF* mice.

Next, we assessed IFN-I production in *TRPV1^cre^-GOF* animals. Cultured DRG neurons from *TRPV1^cre^-GOF* mice exhibited an elevated level of type I IFN-α and -β in the absence of STING stimulation. In addition, ADU-S100 (1 μg/mL) induced a more than 10-fold increase in IFN-β, but not IFN-α, in comparison with untreated neurons (Figure 3, B and C), and ADU-induced increase of both IFN-α and -β was 6-fold larger in cKI animals versus littermate controls (Figure 3, B and C).

We then wanted to determine whether constitutive IFN-I signaling could affect sensory or pain-like behaviors. While baseline mechanical sensitivity was not altered in naive *TRPV1^cre^-GOF* mice (Supplemental Figure 7A), thermal sensitivity as measured by both the hot plate and the Hargreaves test was lower than that of littermate controls in both females and males (Figure 3, D and E). In addition, stereotypical mouse behaviors such as climbing, rearing, eating, drinking, grooming, scratching, and distance moved were normal in *TRPV1^cre^-GOF* cKI animals (Supplemental Figure 7, C–J), indicating that IFN-I production by TRPV1^+^ neurons mainly controlled thermal nociception.

We next evaluated the thermal hyperalgesia of *TRPV1^cre^-GOF* mice after CFA. Although a slightly more pronounced edema was observed at the peak of inflammation in the ipsilateral paw of *TRPV1^cre^-GOF* mice compared with littermate controls (Supplemental Figure 8, A and E), *TRPV1^cre^-GOF* mice exhibited negligible thermal hyperalgesia (Figure 3F). However, mechanical allodynia remained unaffected following CFA (Supplemental Figure 7B). This analgesic effect was not due to a difference in the inflammatory response. Both pro- and antiinflammatory cytokine levels were similar in the inflamed paw of *TRPV1^Cre^-GOF* and *GOF* mice. Moreover, systemic inflammation was not apparent in either mouse group after CFA (Supplemental Figure 8, B–D and F–H; Supplemental Figure 9, A–F; and Supplemental Table 2). To assess whether the type I IFN receptor (IFNAR1) mediated the anti-hyperalgesic effect, we used an anti-IFNAR1 blocking antibody (MAR1). *TRPV1^cre^-GOF* mice received intrathecal injections of MAR1 antibody (100 ng/μL) or IgG control (100 ng/μL) before and 3 days after CFA injection. Inhibition of IFNAR1 by MAR1 antibody restored thermal hyperalgesia in CFA-treated *TRPV1^cre^-GOF* mice (Figure 3G). Because IFNAR1 is expressed in both neuronal and non-neuronal cells of the DRG (11, 22), we assessed the specific role of IFNAR1 expressed in TRPV1^+^ fibers by deleting *Ifnar1* specifically in nociceptors. *TRPV1^cre^-GOF* neonates (P5) received either an i.p. injection of adeno-associated virus (AAV) expressing Cre-inducible *IFNAR1-shRNA* or scramble (Scr) control (Figure 3H). At 6 weeks, we confirmed that neurons of *TRPV1^cre^-GOF* cKI animals were infected with the viral construct (Supplemental Figure 10A). Downregulation of *Ifnar1* transcripts was demonstrated by RNAscope, validating the efficacy of the *IFNAR1-shRNA* vector (Supplemental Figure 10B). Depletion of *Ifnar1* in *TRPV1^cre^-GOF* neurons restored normal thermal sensitivity (Figure 3I). Importantly, mice treated with *IFNAR1-shRNA* displayed thermal hyperalgesia compared with *IFNAR1-Scr* mice after CFA injection (Figure 3J). Because TRPV1 is expressed in a wider population of neurons during development, we addressed a potential compensatory mechanism of *Ifnar1* knockdown in pups by delivering the AAV in adult *TRPV1^cre^-GOF* mice (Figure 3K). As found with the injected pups, thermal hypersensitivity was restored in adult *TRPV1^cre^-GOF* mice that received the *IFNAR1-shRNA* (Figure 3L). Overall, these results show that activation of neuronal STING might promote resolution of pain after inflammation, at least in part via autocrine IFN-I/IFNAR1 signaling.

### ISGs reduce intrinsic excitability of nociceptors.

**Figure 4:**
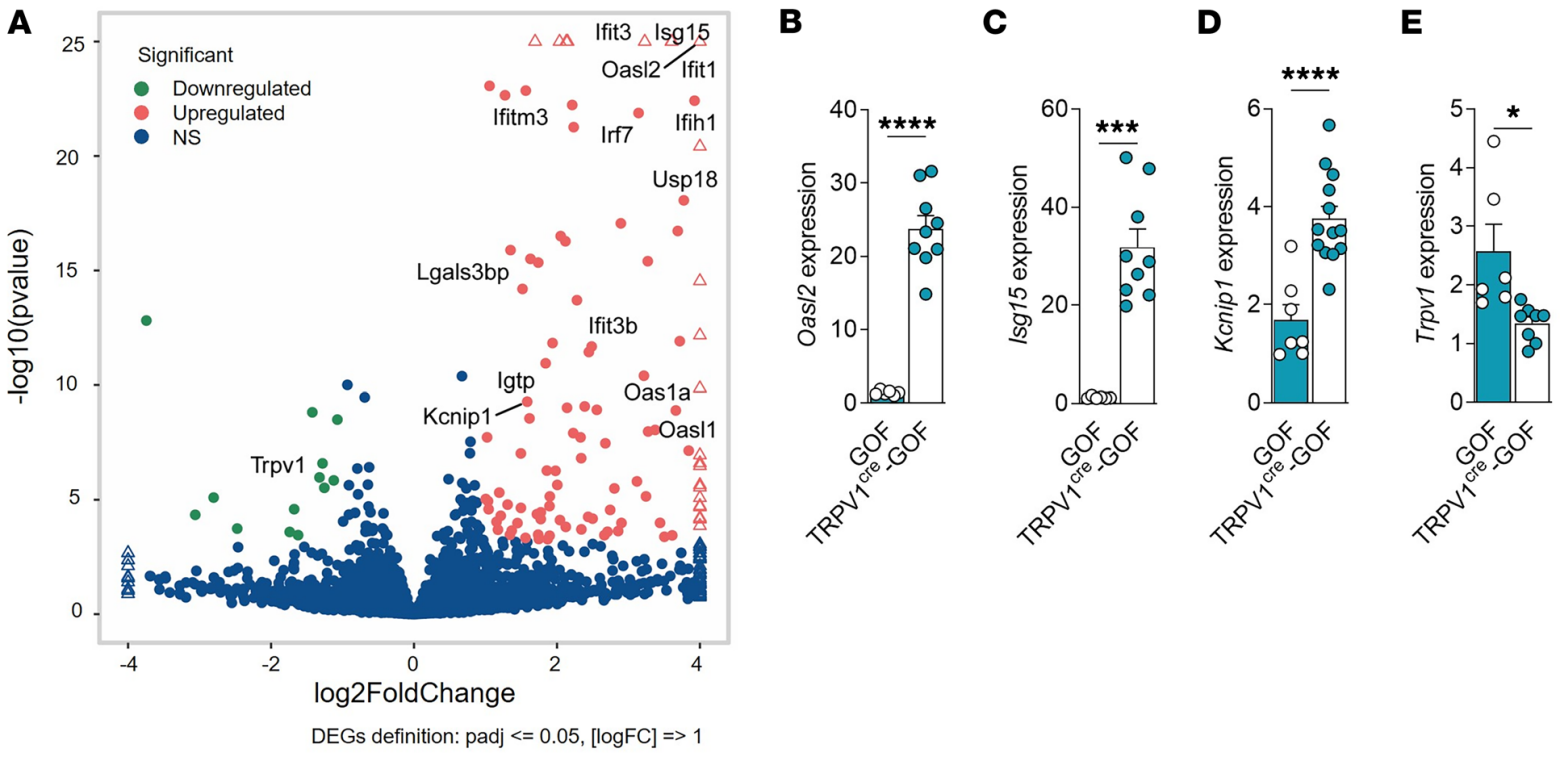
Nociceptor-specific *STING-N154S* gain of function induces IFN-I production and expression of IFN-stimulated genes in DRGs. (**A**) Volcano plot representation of genes regulated in naive *TRPV1^cre^-GOF* cKI mice. Genes that pass a threshold of log_1.5_ fold change in differential expression analysis are colored green when they are downregulated and red when they are upregulated. (**B**) *Oasl2* expression in lumbar DRG neurons of naive *GOF* (*n* = 6) and *TRPV1^cre^-GOF* (*n* = 9) mice. (**C**) *Isg15* expression in lumbar DRG neurons of naive *GOF* (*n* = 6) and *TRPV1^cre^-GOF* (*n* = 9) mice. (**D**) *Kchip1* expression in lumbar DRG neurons of naive GOF (*n* = 7) and *TRPV1^cre^-GOF* (*n* = 13) mice. (**E**) *Trpv1* expression in lumbar DRG neurons of naive *GOF* (*n* = 6) and *TRPV1^cre^-GOF* (*n* = 8) mice. Statistical analysis was performed using *t* test (**B**, **D**, and **E**; *P < 0.05, ****P < 0.0001) or Mann-Whitney (**C**; ***P < 0.001).

**Figure 5:**
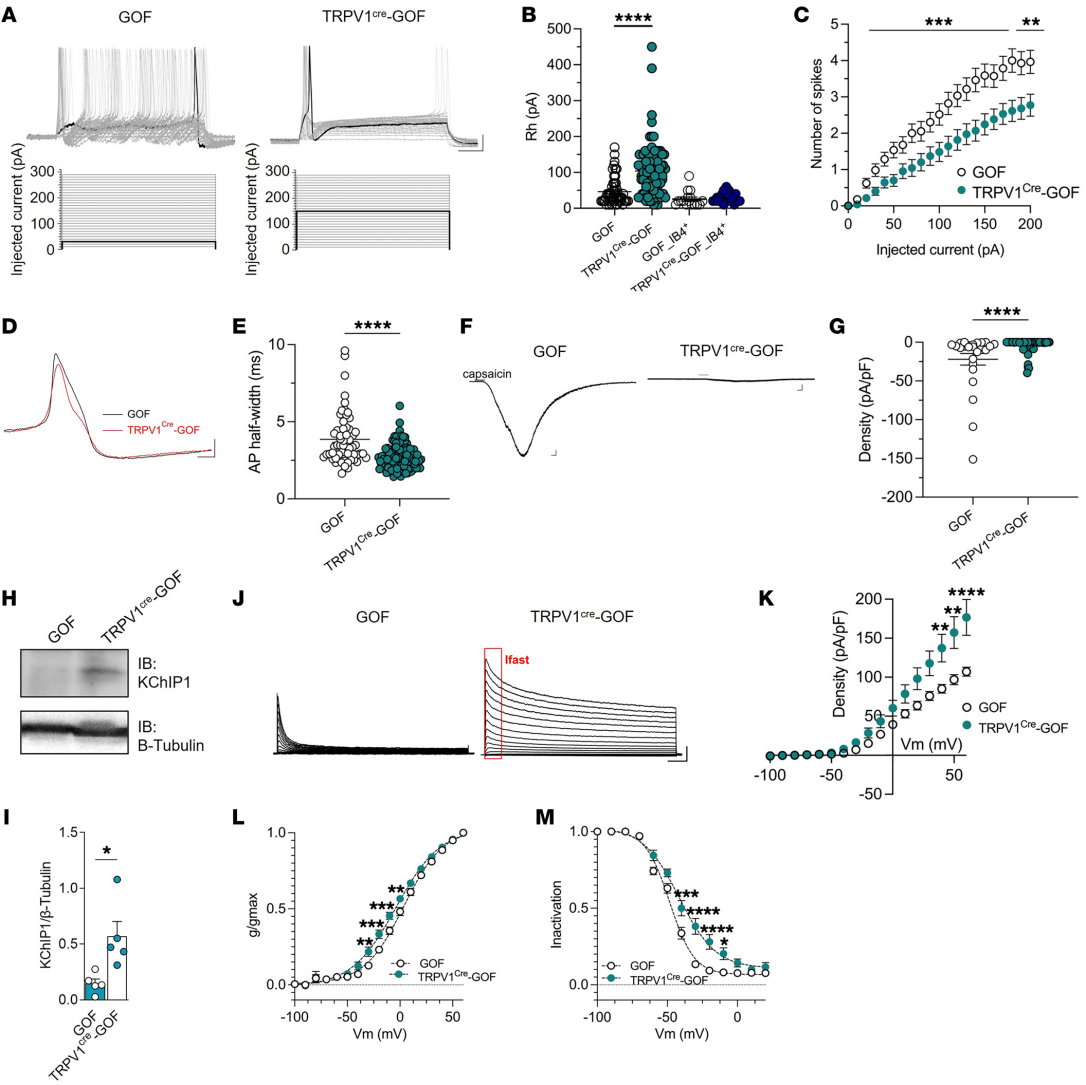
ISGs alter nociceptor properties through TRPV1 downregulation and KChIP1 expression. (**A**) Representative current clamp recording of evoked action potentials (APs) recorded in TRPV1 neurons (top). Cells were injected with a 500-millisecond current pulse with an increment of 10 pA and an interval of 5 seconds (protocol, bottom). The highlighted black line indicates the current amplitude that induces the first AP. Scale bars: 20 mV/50 ms. (**B**) Rheobase data recorded in TRPV1 and non-peptidergic (IB4^+^) neurons from *GOF* (*n* = 61 and *n* = 16, respectively) and *TRPV1^cre^-GOF* mice (*n* = 101 and *n* = 22, respectively). (**C**) Number of spikes as a function of injected current in TRPV1 neurons. (**D**) Representative APs recorded in TRPV1 neurons from *GOF* (*n* = 61) and *TRPV1^cre^-GOF* (*n* = 101) mice. Scale bars: 20 mV/50 ms. (**E**) AP half-width recorded in **D**. (**F**) Representative currents induced by capsaicin (100 nM) in TRPV1 neurons. Scale bars: 200 pA/10 s. (**G**) Current density evoked by capsaicin in TRPV1 neurons from *GOF* (*n* = 25) and *TRPV1^cre^-GOF* (*n* = 70) mice. (**H**) Representative Western blot of KChIP1 protein level. Three independent experiments were performed. (**I**) Quantification of KChIP1 protein level in lumbar DRG from naive *GOF* (*n* = 5) and *TRPV1^cre^-GOF* (*n* = 5) mice. (**J**) Representative outward potassium currents recorded in response to voltage steps in TRPV1 neurons. Scale bars: 1 nA/100 ms. (**K**) Average current-voltage relationship from neurons recorded in **J** (*GOF*, *n* = 10; *TRPV1^cre^-GOF*, *n* = 17). (**L** and **M**) Steady-state activation (**L**) and inactivation (**M**) from neurons recorded in **J** (activation: *GOF*, *n* = 17; *TRPV1^cre^-GOF*, *n* = 17; inactivation: *GOF*, *n* = 18; *TRPV1^cre^-GOF*, *n* = 25). All steady-state plots were fitted with Boltzmann functions to derive *V*_½_ and *k* values. Statistical analysis was performed using Kruskal-Wallis followed by Dunn’s post hoc test (**B**; ****P < 0.0001), 2-way ANOVA followed by Tukey’s post hoc test (**C** and **K**–**M**; *P < 0.05, **P < 0.01, ***P < 0.001, ****P < 0.0001), and Mann-Whitney test (**E** and **G**) or *t* test (**I**; *P < 0.05, ****P < 0.0001).

To evaluate the effect of IFN-I signaling on nociceptors, we conducted bulk RNA-Seq on DRGs from *TRPV1^cre^-GOF* mice. Comparative transcriptomic profiling showed a marked IFN signature associated with a dozen downregulated genes and more than 100 upregulated ISGs in DRGs from naive *TRPV1^cre^-GOF* cKI mice, compared with *GOF* littermate controls (Figure 4A and Supplemental Table 3). Among upregulated genes, we found classical ISGs known to trigger protective defense mechanisms against pathogens or tumors: *Oasl*, *Ifitm3*, *Isg15*, *Ccl5*, and *Usp18* mRNAs. These findings indicated that constitutive activation of STING effectively promoted ISG expression in TRPV1 nociceptors, thus validating our *TRPV1^cre^-GOF* cKI model to study the IFN gene responses in these neurons. While the function of ISGs enables host defense, and allows cells to recover to normal function, only a handful of these genes have been studied in detail (10), and none of them in the context of neuronal plasticity. Interestingly, nociceptor-specific ion channels, including *Trpv1*, *Trpa1*, and *Trpc3*, were downregulated in *TRPV1^cre^-GOF* DRGs, whereas the A-type Kv channel–regulating protein (*Kchip1*), known to suppress excitability, was upregulated (Figure 4A). Gene expression quantification by quantitative reverse transcription PCR confirmed the increase in *Oasl2* (Figure 4B), *Isg15* (Figure 4C), and *Kcnip1* (KChIP1) transcripts (Figure 4D) in *TRPV1^cre^-GOF* neurons, whereas *Trpv1* mRNA was significantly reduced (Figure 4E), corroborating the reduction of TRPV1^+^ neurons in *TRPV1^cre^-GOF* mice (Supplemental Figure 4C). To investigate the functional impact of ISGs on nociceptors, we performed electrophysiological recordings of TdTomato^+^ *TRPV1^cre^-GOF* nociceptors (Figure 5A). While the action potential (AP) amplitude (Supplemental Figure 11A) and the resting membrane potential (Supplemental Figure 11B) were unchanged, an increase in rheobase (Figure 5, A and B) and input resistance (Supplemental Figure 11C) was measured in *TRPV1^cre^-GOF* neurons compared with littermate controls (*GOF*). In contrast, the rheobase of non-peptidergic IB4^+^ neurons from *TRPV1^cre^-GOF* mice was similar to that of IB4^+^ neurons isolated from littermate controls (Figure 5B and Supplemental Figure 12, A and B), suggesting that IFN-induced hypoexcitability was specific to TRPV1 neurons in *TRPV1^cre^-GOF* mice. Similarly, the number of APs evoked by depolarizing current was lower in *TRPV1^cre^-GOF* compared with littermate control neurons (Figure 5C). Lastly, looking at the AP half-width, *TRPV1^cre^-GOF* mice had a shorter AP duration, indicating larger hyperpolarizing K^+^ currents in *TRPV1^cre^-GOF* neurons (Figure 5, D and E). Given that *Trpv1* gene expression was reduced in *TRPV1^cre^-GOF* DRGs (Figure 4E and Supplemental Figure 4C), we assessed capsaicin-evoked currents (100 nM) in isolated DRG neurons. We found a pronounced decrease in TRPV1 current density (Figure 5, F and G). Consistent with the observation of *Kchip1* upregulation by sequencing (Figure 4D), we found that the KChIP1 protein level was augmented in *TRPV1^cre^-GOF* lumbar DRGs (Figure 5, H and I). As KChIP1 serves as a specific β-subunit for Kv4 channels (26, 27), we analyzed the electrophysiological properties of A-type K^+^ currents in *TRPV1^cre^-GOF* neurons. Notably, the Kv4-mediated A-type current was larger in *TRPV1^cre^-GOF* neurons (Figure 5, J and K), supporting an increase in Kv4 channel opening or surface trafficking, induced by KChIP1 upregulation. Accordingly, the steady-state activation of A-type Kv4 channels was shifted to more hyperpolarized potentials (Figure 5L), while the steady-state inactivation was delayed in *TRPV1^cre^-GOF* neurons (Figure 5M), indicating a regulation of Kv4 channel gating by KChIP1. Taken together, our results show that ISGs regulate the expression of ion channels and channel-interacting proteins to enhance the threshold of nociceptor activation and reduce the excitability of nociceptive neurons.

### IFNAR1 depletion normalizes ion channel expression and electrophysiological properties.

**Figure 6:**
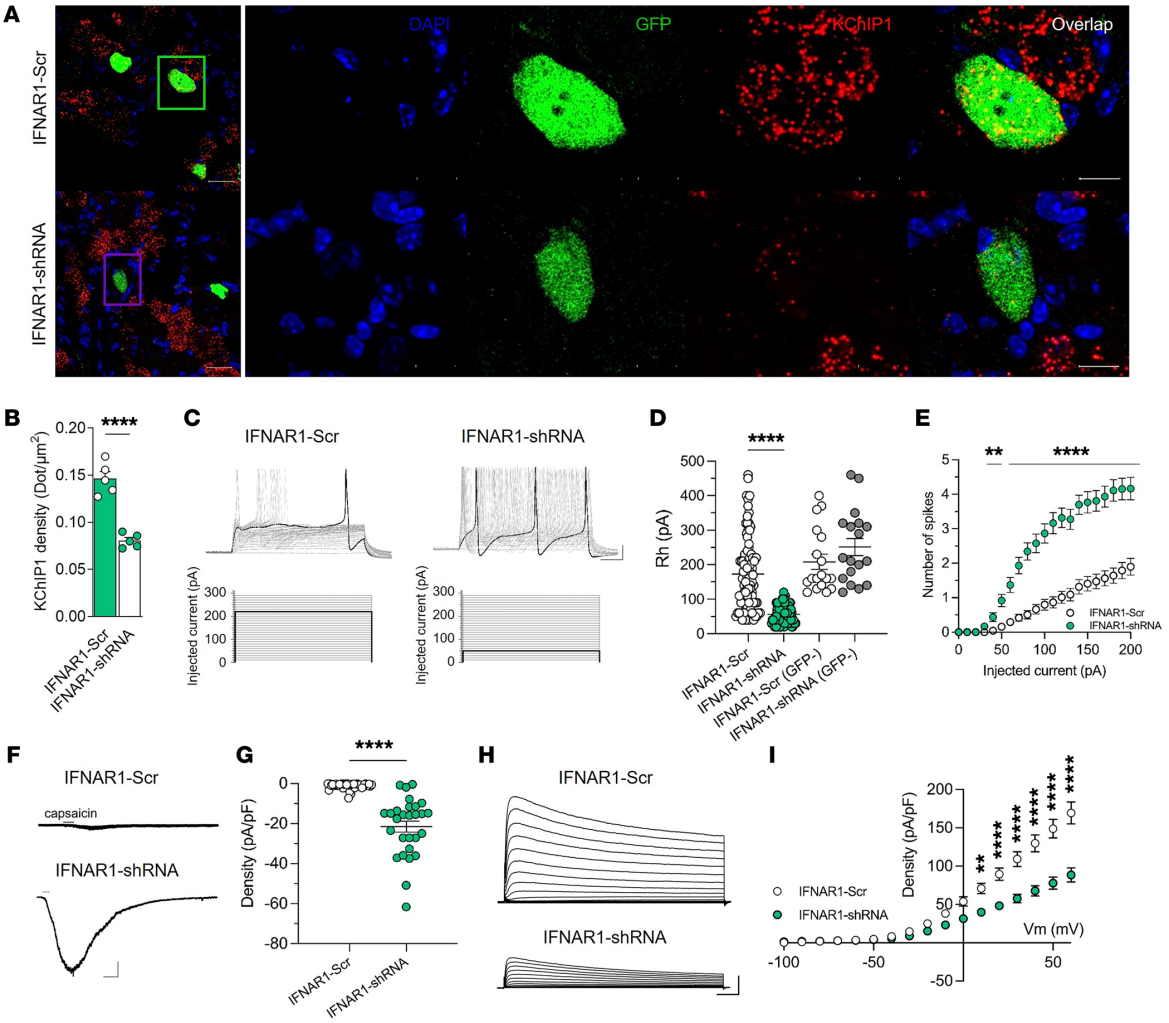
IFNAR1 depletion in nociceptors restores electrophysiological properties. (**A**) Confocal image of TRPV1 neurons from *TRPV1^Cre^-GOF* mice injected with *DIO-Scr-shRNA* (*n* = 5) or *DIO-IFNAR1-shRNA* AAVs (*n* = 5). Images represent DAPI staining, AAV-GFP expression (green), and *Kchip1* transcripts (red) by RNAscope. Scale bars: 25 μm, and 10 μm on cropped images. (**B**) Quantification of *Kchip1* density measured by the number of transcripts represented by dots per surface unit in AAV-infected TRPV1 neurons. (**C**) Representative current clamp recording of TdTomato^+^/GFP^+^ TRPV1 neurons from *TRPV1^Cre^-GOF* mice infected with *DIO-Scr-shRNA* or *DIO-IFNAR1-shRNA* AAVs (top). Cells were injected with 500-millisecond current pulses with an increment of 10 pA and an interval of 5 seconds (protocol, bottom). The highlighted black line indicates the current amplitude that induces the first AP. Scale bars: 20 mV/50 ms. (**D**) Measurement of rheobase in infected (GFP^+^) and non-infected (GFP^–^) TRPV1 neurons (TdTomato^+^) recorded in **C**. Data are presented as dot plots with mean values (*IFNAR1-Scr* neurons, *n* = 89; *IFNAR1-shRNA*–infected neurons, *n* = 80; *IFNAR1-Scr* neurons, *n* = 18; *IFNAR1-shRNA*–non-infected neurons, *n* = 18). (**E**) Number of spikes evoked by injected current in TRPV1 (TdTomato^+^) and AAV-infected (GFP^+^) neurons. (**F**) Representative capsaicin-evoked current (100 nM) in TdTomato^+^/GFP^+^ TRPV1 neurons from *TRPV1^Cre^-GOF* mice infected with *IFNAR1-Scr* and *IFNAR1-shRNA* AAVs. Scale bars: 200 pA/20 s. (**G**) Current density evoked by capsaicin in cells represented in **F** (*IFNAR1-Scr* neurons, *n* = 41; *IFNAR1-shRNA* neurons, *n* = 28). (**H**) Representative outward potassium currents recorded in response to voltage steps in *TRPV1^Cre^-GOF* neurons infected with *IFNAR1-Scr* and *IFNAR1-shRNA* AAVs. Scale bars: 1 nA/100 ms. (**I**) Average current-voltage relationship in TRPV1 neurons from *TRPV1^Cre^-GOF* mice infected with *IFNAR1-Scr* (*n* = 31) or *IFNAR1-shRNA* AAVs (*n* = 25). Statistical analysis was performed using *t* test (**B**) or Mann-Whitney test (**D** and **G**; ****P < 0.0001) and 2-way ANOVA with Tukey’s post hoc test (**E** and **I**; **P < 0.01, ****P < 0.0001).

To understand whether the electrophysiological phenotype of *TRPV1^cre^-GOF* neurons was mediated by IFN-I/IFNAR1 signaling, *TRPV1^cre^-GOF* neonates (P5) received an i.p. injection of Cre-inducible AAV-GFP expressing either *IFNAR1-shRNA* (1 × 10^13^ genome copies [GC]/mL) or scramble control (1 × 10^13^ GC/mL). Depletion of *Ifnar1* in *TRPV1^cre^-GOF* neurons restored normal *Kchip1* expression (Figure 6, A and B). Then, DRG neurons were dissociated, and electrophysiological recording of AAV-infected or non-infected *TRPV1^cre^-GOF* neurons was performed (Figure 6C). Notably, *IFNAR1-shRNA*–infected neurons had a much lower rheobase compared with non-infected or *IFNAR1-Scr*–infected *TRPV1^cre^-GOF* neurons (Figure 6D). This was consistent with an increase in evoked AP firing observed in *IFNAR1-shRNA*–infected cells (Figure 6E). In addition, *Ifnar1* depletion was able to restore TRPV1 current density (Figure 6, F and G) and reduce Kv4-mediated A-type current (Figure 6, H and I) induced by IFN-I.

### KChIP1/Kv4 interaction promotes the anti-nociceptive effect of ISGs.

**Figure 7:**
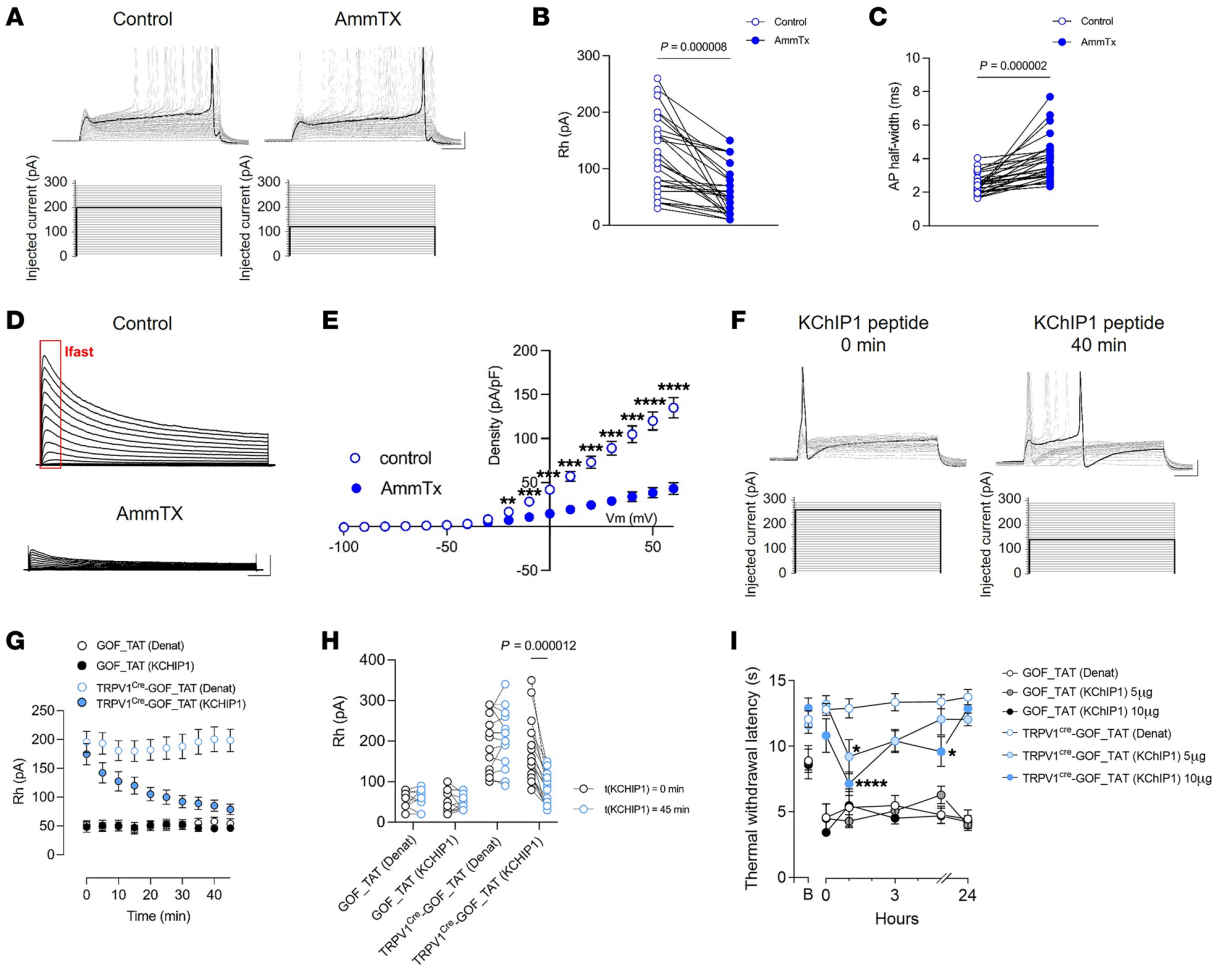
KChIP1/Kv4 interaction promotes the anti-nociceptive effect of ISGs. (**A**) Representative current clamp recording of *TRPV1^cre^-GOF* neurons, in control condition and after AmmTx3 (1 μM) application (top). Cells were injected with 500-millisecond current pulses with an increment of 10 pA and an interval of 5 seconds (protocol, bottom). The highlighted black line indicates the current amplitude that induces the first AP. Scale bars: 20 mV/50 ms. (**B** and **C**) Measurement of rheobase (**B**) and AP half-width (**C**) induced by AmmTx3 application in *TRPV1^cre^-GOF* neurons (*n* = 29 neurons). (**D**) Representative outward potassium currents recorded in response to voltage steps in *TRPV1^cre^-GOF*, in control condition and after AmmTx3 application. Scale bars: 1 nA/100 ms. (**E**) Average current-voltage relationship obtained from the cells recorded in **D** (*n* = 9). (**F**) Representative current clamp recording of *TRPV1^cre^-GOF* neurons treated with a TAT-conjugated KChIP1 peptide for 40 minutes (top). Cells were injected with 500-millisecond current pulses with an increment of 10 pA and an interval of 5 seconds (protocol, bottom). The highlighted black line indicates the current amplitude that induces the first AP. Scale bars: 20 mV/50 ms. (**G**) Time-dependent effect of TAT-conjugated KChIP1 versus denatured control peptide on the rheobase of *TRPV1^Cre^-GOF* neurons (*n* = 15 and 17, respectively) and *GOF* control neurons (*n* = 10 and 11, respectively). (**H**) Measurement of rheobase at *t* = 0 and 45 minutes after KChIP1 exposure or denatured peptide in *TRPV1^cre^-GOF* neurons (TAT-Denat, *n* = 15; TAT-KChIP1, *n* = 17) or GOF neurons (TAT-Denat, *n* = 10; TAT-KChIP1, *n* = 12). (**I**) Measurement of thermal withdrawal latency in hind paws of both CFA-treated *GOF* and *TRPV1^cre^-GOF* mice treated with 5 μg (*n* = 7, 7) or 10 μg (*n* = 6, 7) KChIP1 blocking peptide or its denatured control (*n* = 6, 7) at day 3 after CFA injection. Statistical analysis was performed using paired *t* test (**B**, **C**, and **H**) and 2-way ANOVA followed by Tukey’s post hoc test (**E** and **I**; *P < 0.05, **P < 0.01, ***P < 0.001, ****P < 0.0001).

Our results indicate that *Kcnip1* (KChIP1) is a key IFN-regulated gene in response to nociceptor STING activation. Since we observed an increase in A-type K^+^ current and KChIP1 is an integral subunit component of the native Kv4 channel complex, we wondered how the Kv4-KChIP1 subunit complex could contribute to the anti-nociceptive effect of ISGs. To investigate this, we first tested AmmTx3, a specific Kv4 channel blocker. In *TRPV1^cre^-GOF* nociceptors, AmmTx3 (1 μM) decreased the high rheobase (Figure 7, A and B), prolonged the AP duration (Figure 7C), and reduced A-type Kv current density (Figure 7, D and E), implying an IFN-I–dependent upregulation of A-type K^+^ current induced by the interaction between KChIP1 and Kv4.

To evaluate this hypothesis, we used a TAT-conjugated KChIP1 interfering peptide to disrupt the KChIP1-Kv4.3 or KChIP1-Kv4.1 subunit complex. Intrapipette administration of the KChIP1 peptide (20 nM) in *TRPV1^cre^-GOF* neurons led to a gradual reduction of the high rheobase over time (Figure 7, F and G). The effect of KChIP1 peptide was significant after 20 minutes and reached saturation at 45 minutes after infusion. In contrast, no time-dependent inhibition of the rheobase was observed with a heat-denatured KChIP1 peptide or in the absence of KChIP1 upregulation (*GOF* littermate neurons) (Figure 7, G and H). Having validated the efficacy of the KChIP1-Kv4–disrupting peptide, we investigated whether we could target the subunit association in *TRPV1^cre^-GOF* mice. Intrathecal infusion of KChIP1 peptide, at 5 and 10 μg, revealed thermal hyperalgesia for 3 hours in *TRPV1^cre^-GOF* mice following intraplantar CFA (Figure 7I). The same peptide, administered to GOF littermates, did not disrupt CFA-induced hyperalgesia. Together, our results establish that the KChIP1-Kv4 interaction is responsible for the IFN-I–induced anti-nociception, downstream of neuronal STING.

## Discussion

In this study, we have identified neuronal IFN-I as a critical regulator of ion channels and channel-interacting proteins in nociceptors during the resolution of inflammation. Our findings highlighted KChIP1 and TRPV1 as downstream effectors of IFN-I, playing a pivotal role in the resolution of hyperalgesia and the restoration of normal excitability following sensitization.

**Figure 8:**
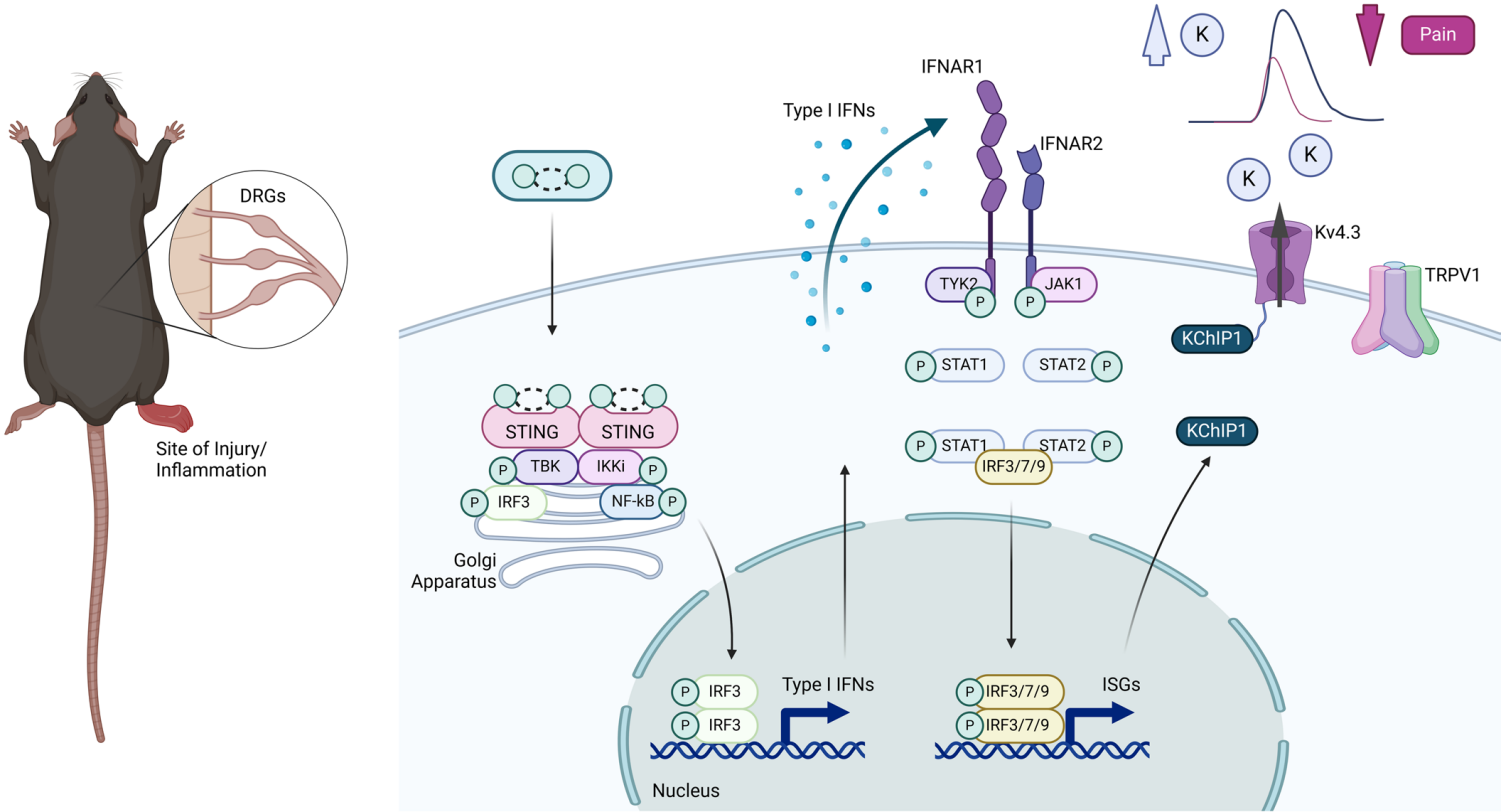
Schematic representation of pain-resolving effects of the STING/IFN-I pathway in inflammatory pain models. Upregulation of nociceptor STING during inflammation stimulates TANK-binding kinase 1 (TBK1). TBK1 phosphorylates IFN regulatory factor 3 (IRF3), which controls the production of type I IFN, including IFN-β. Type I IFNs (IFN-I) bind to IFN-α/β receptor (IFNAR) on TRPV1 nociceptors to initiate the transcriptional regulation of hundreds of IFN-regulated genes (IRGs), which reduces heat hyperalgesia (TRPV1 downregulation) and nociceptor excitability (KChIP1 upregulation), promoting pain resolution.

We show that tissue inflammation leads to an increase in STING, a cytosolic DNA sensor, within nociceptors. Constitutive activation of STING triggers TBK1 signaling and IFN-β production, which is sufficient to induce an antiviral ISG response, ultimately reprogramming the gene expression profiles of nociceptors. Consequently, this process results in a nociceptor-specific IFNAR1-dependent reduction in excitability and inflammatory hyperalgesia (Figure 8).

Our findings not only highlight STING as a valuable marker for nociceptor sensitization but also underscore the importance of IFN-regulated genes, particularly *Kchip1*, in the control of persistent pain conditions.

Nociceptors are specialized sensory neurons that detect a wide range of danger signals, including those from pathogens or the host, through the expression of pattern recognition receptors such as *Sting* (8). The cGAS/STING pathway, which detects cytosolic DNA, is evolutionarily conserved among diverse animal lineages (8, 23). Consistent with previous studies (8, 22), we found expression of *Sting* in nociceptors of both mouse and human DRG neurons. We demonstrate that during inflammation, the expression and activation of STING were upregulated in TRPV1^+^ neurons, which are responsible for detecting and responding to noxious stimuli. Certain pathogens, such as SARS-CoV-2, have evolved mechanisms to suppress sensory neurons related to smell and taste, and not all viral infections exhibit symptoms. While further investigation is needed to elucidate whether this nociceptor-specific STING/IFN-I signaling contributes to antiviral responses, it may facilitate the resolution of inflammation, leading to the downregulation of pain once the viral infection has been cleared.

STING responds to both self and non-self insults. While complete Freund’s adjuvant (CFA), which contains a suspension of heat-killed mycobacteria, enhances IFN-I production, heat-inactivated *Mycobacterium tuberculosis* does not have the same effect (28, 29). However, the adjuvant activity of CFA stems from its ability to stimulate a local innate immune response, leading to a delayed hypersensitivity reaction and an intense inflammatory response at the site of injection (30). Interestingly, NF-κB signaling in response to cytokines such as IL-6 or IL-1β enhances STING-mediated immune responses by inhibiting the microtubule-mediated trafficking of STING from the Golgi apparatus to lysosomes for degradation (31). Additionally, a burst of mitochondrial reactive oxygen species (ROS), during inflammation, can trigger the opening of the mitochondrial permeability transition pore (mPTP), resulting in the extracellular release of mitochondrial DNA (mtDNA). Leakage of mitochondrial components into the cytosol acts as damage-associated molecular patterns, recruiting cGAS-STING and triggering an immune response (32). Indeed, the production of IL-1β induces the release of mtDNA, which activates cGAS-STING and enhances IFN-I production (33).

The role of the STING/IFN-I pathway in nociception has been a subject of debate in recent years. While administration of STING agonists through the central route causes acute analgesia in naive animals, peripheral STING agonists or IFN-I injection promotes mechanical allodynia (11, 14, 34). Furthermore, STING activation in response to exogenous ligands acutely reduces mechanical allodynia in various neuropathic pain models through IFN-I/IFNAR1 signaling (11, 12). To investigate the role of IFN-I specifically in neurons, we used a gain-of-function approach by expressing human N154S *Sting* mutation in TRPV1 nociceptors, resulting in constitutive IFN-I production. Our findings indicate that IFN-I/IFNAR1 signaling regulates thermal but not mechanical sensitivity in both naive and inflammatory conditions. These results support previous studies highlighting the importance of TRPV1 neurons in thermal sensitivity and hyperalgesia. Accordingly, ablation of TRPV1 neurons or their central terminals in the spinal cord using chemical or genetic methods reduces thermal sensitivity, while mechanical sensitivity remains unaffected, even after sensitization by inflammation or nerve injury (35, 36). Additionally, chemogenetic inhibition of TRPV1 neurons using the designer receptors exclusively activated by designer drugs (DREADD) system does not alter mechanical responses (37). Notably, Yu and colleagues have identified 2 peptidergic populations of nociceptors in human DRGs that coexpress TRPV1 and TRPA1, but not PIEZO (38), suggesting that, at least in humans, TRPV1 neurons predominantly respond to noxious thermal and chemical stimuli, while their sensitivity to mechanical stimuli might be relatively lower.

Strikingly, while the development of thermal hypersensitivity was prevented in mice with nociceptor-specific *hSTING* mutation, the edema caused by CFA was slightly enhanced. Gain-of-function mutations in STING, including the N154S mutation, underlie a type I interferonopathy called STING-associated vasculopathy with onset in infancy (SAVI). SAVI is a severe disease characterized by early-onset systemic inflammation, skin vasculopathy, and interstitial lung disease. SAVI patients exhibit high levels of C-reactive protein (39) and upregulated NF-κB–related protein (e.g., IL-6) (40). Neuronal overproduction of IFN-I may directly impact the immune system in the periphery. IFN-I has been shown to indirectly inhibit tumor growth by acting on immune cells. Natural killer cells, the first line of defense against infections and tumors, depend on type I IFNs for maturation, activation, and homeostasis in the tumor microenvironment (TME) (41, 42). IFN-I also promotes maturation and migration of dendritic cells toward lymph nodes, where their activity is enhanced through the cross-presentation of tumor-associated antigens to CD8^+^ T cells (43–45). Additionally, IFN-I may enhance a proinflammatory environment by inhibiting Treg proliferation, function, and recruitment, as observed in the TME (46–48). Further studies are needed to elucidate the relative role of neuronal IFN-I in immune responses to inflammation.

Our findings also highlight IFNAR1 in the STING/IFN pathway. IFNAR is widely expressed in peptidergic nociceptors and their central terminals in the spinal dorsal horn (14, 49). The localization of IFNAR1 in the presynaptic terminals suggests a role for STING/IFN-I in regulating neurotransmitter release. Indeed, Liu and colleagues reported that IFNAR activation inhibits excitatory synaptic transmission in somatostatin-positive excitatory neurons, by suppressing glutamate release from presynaptic terminals (49). Although *Ifnar1* expression in nociceptors is prominent, non-neuronal IFNAR1 may also contribute to the anti-nociceptive effects of IFN-I. Emerging evidence suggests that spinal cord microglia and astrocytes play important roles in the regulation of pain hypersensitivity following tissue and nerve injuries (50, 51). Intrathecal administration of poly I:C, a synthetic dsRNA, increases the expression of *Irf7* and *Irf9*, indicating that both astrocytes and microglia respond to IFN-I (52, 53). Nevertheless, using nociceptor-specific AAVs to knock down *Ifnar1* in *TRPV1^cre^-GOF* mice, we found that neuronal type I IFNs suppressed nocifensive behaviors through their cognate IFNAR1 receptor on TRPV1 nociceptors.

Furthermore, comparative transcriptomic profiling of DRGs revealed a marked IFN signature associated with downregulated genes and upregulated ISGs in response to STING activation. Among the ISG repertoire, known antiviral and antitumor genes were identified, including *OASL*, *EIF2AK2* (PKR), *Irf7*, *Ifit1*, *Ddx58*, *Usp18*, and *ISG15*. Interestingly, eukaryotic initiation factor 2 (eIF2), a key effector of cellular stress responses, participates in the reduction of protein translation via phosphorylation by PKR. This reduction in protein translation allows cells to conserve energy and modify gene expression to effectively manage stress conditions (54). While exogenous IFN-I does not result in PKR/eIF2α activation in DRGs (14), there is evidence suggesting the involvement of this pathway in the regulation of thermal sensitivity (14, 55). Neurons may utilize the PKR/eIF2α pathway to modulate ion channel expression, including TRPV1 and Kv4 channels, and facilitate the resolution of inflammatory hyperalgesia.

One of the ISGs identified in *TRPV1^cre^-GOF* DRGs was the *Kcnip1* gene, which encodes the KChIP1 auxiliary subunit of voltage-gated potassium (Kv) channels. In line with our findings, *Kcnip1* is a candidate gene involved in heat pain and has been identified as being enriched in TRPV1^+^ neurons (56–58). KChIPs and transmembrane dipeptidyl peptidase–like proteins (DPPs) are part of the Kv4 channel complex that generates A-type K^+^ currents (59–61) and inhibits nociceptor excitability below the threshold for AP generation (62). KChIP1 modulates the trafficking, voltage dependence, and inactivation kinetics of Kv4, thereby affecting the firing patterns and excitability of sensory neurons (59, 63, 64). While Kv4.1 channels are expressed in all DRG neurons, Kv4.3 is predominantly found in small-diameter neurons (65–67), specifically non-peptidergic IB4^+^ and peptidergic TRPV1^+^ neurons but not CGRP^+^ neurons (65, 67–69). Notably, Kv4.3 channels are distributed across the entire cell surface of sensory neurons, including their peripheral endings in the skin (70, 71). Given that Kv4.3 operates within the subthreshold voltage range, this channel may function in the cutaneous endings to inhibit AP initiation in response to non-noxious stimuli. Additionally, Kv4.3 channels exhibit high temperature sensitivity within a narrow hyperpolarized voltage range (72), a property that could potentially be influenced by the level of KChIP1 expression. Our findings demonstrate that the constitutive activation of STING and the production of IFN-I specifically reduce the excitability of TRPV1 neurons while leaving the properties of IB4^+^ neurons unchanged. This supports a model in which IFN-induced *Kchip1* expression reduces AP initiation in TRPV1^+^ nociceptors, thereby increasing the activation threshold of nociceptors to noxious stimuli.

Overall, our results describe a nociceptor-specific antiviral pain-resolving ISG response, which alters the expression of the ion channel–associated protein *Kchip1* and nociceptive ion channels such as *Trpv1*. This STING-mediated response helps counteract inflammatory hyperexcitability and restores the nociceptor threshold after inflammation. This modulation may occur through the PKR/eIF2α pathway, which has been previously described to control proteostasis in nociceptors (14, 54). While recent work has explored the role of STING in pain sensitivity in the context of neuropathic pain, our findings show that inflammation leads to an upregulation of STING in nociceptors. This upregulation is associated with an IFN-I response that promotes resolution of inflammatory pain. Furthermore, our study provides important mechanistic insights into the ion channel and gene expression changes relevant to STING activation in the context of inflammation. Further studies are needed to elucidate the downstream signaling pathways and transcriptional changes that mediate the effects of IFN-I in nociceptors. Additionally, investigating the role of IFN-I/IFNAR1 signaling in other types of sensory neurons, such as non-peptidergic nociceptors, and in different pain models will be crucial for understanding the broader implications of this signaling pathway in pain resolution.

By identifying STING-ISGs as a key pain-resolving response that turns down nociceptor excitability in inflammatory pain conditions, we have added an important innate immune molecule to the core list of pattern recognition receptors that regulate the integration of pain signals in primary afferent neurons (11, 12, 73–75). Harnessing the power of IFN-I and ISGs to alleviate hyperexcitability of nociceptors may provide a unique therapeutic arsenal for managing inflammatory pain conditions.

## Methods

Further information can be found in Supplemental Methods.

### Sex as a biological variable

Age- and sex-matched 6- to 8-week-old littermate mice were used for experiments.

### Mice

C57BL/6J (strain 000664) and *B6.Rosa26-TdTomato* reporter mice (*ai14* strain, 007914) were purchased from The Jackson Laboratory. *Nav1.8-Cre* transgenic (Tg) mice (also known as *SNS-Cre* mice) were from Rohini Kuner’s laboratory (University of Heidelberg, Heidelberg, Germany) and previously published (18). *Nav1.8-Cre* Tg mice were bred with *ai14* mice to generate *Nav1.8-Cre/TdTomato* mice. The *STING^–/–^* (*Tmem173gt* C57BL/6J) and the *flox-GOF* (*B6N.CAG-loxP-STOP-loxP-constitutively-active-hSTING-N154S mutant-IRES-TdTomato*) mice were provided by F. Jirik (University of Calgary, Calgary, Alberta, Canada), and the *TRPV1-Cre* and *TRPV1-pHluorin* mice (21) were bred at the University of Calgary Animal Resource Center. All mice were genotyped with the primers reported in Supplemental Table 4. To study the gain of function of STING in TRPV1 neurons, *TRPV1-Cre^+/−^* mice were bred with *flox-STING-hN154S* mice to generate mice with TRPV1 neurons expressing GOF (*TRPV1-Cre^+/–^ hSTING-N154S^+/–^* or *TRPV1^cre^-GOF*) and control littermates (*TRPV1-Cre^–/–^ hSTING-N154S^+/–^* or *GOF*). To reduce the risk of bias that can be caused by an awareness of group assignment, single-blind experiments were performed. All mice were housed under standard conditions with both drinking water and food available ad libitum.

### Treatment with chemicals, antibodies, and viral constructs

#### Complete Freund’s adjuvant injections.

Mice were anesthetized by isoflurane and injected in the right hind paw with 20 μL of complete Freund’s adjuvant (CFA; purchased from Sigma-Aldrich) using a Hamilton syringe fitted with a 30-gauge needle. Paw edema was measured with an electronic caliper.

#### AAV injections.

Adeno-associated virus (AAV2-PHP.S) was used for expression of *IFNAR1-shRNA1–3* in *TRPV1^cre–^GOF* cKI animals. *AAV2-CW3SL-CAG-DIO-IFNAR1-shRNA1–3-eGFP* and *AAV2-CW3SL-CAG-DIO-IFNAR1-Scr-eGFP* (1 × 10^13^ GC/mL) were produced at the University of Calgary. The shRNAs were designed using the SplashRNA algorithm (76). Newly born pups (P5) were given 10 μL (i.p.) of the viral construct at 1 × 10^13^ GC/mL using a Hamilton syringe connected to a 30-gauge syringe tip (Becton Dickinson). Pups were returned to their parent cages and weaned after 21 days. AAVs were administered intrathecally in adult mice (6 weeks old). Briefly, mice were anesthetized by isoflurane, the dorsal fur of each mouse was shaved, the spinal column was arched, and a 30-gauge needle was inserted into the subarachnoid space between the L4 and L5 vertebrae. Intrathecal injections of 10 μL were delivered over a period of 5 seconds.

#### Neutralizing antibodies.

Anti–mouse IFN-β neutralizing antibody (300 ng/mouse; PBL Assay Science, 32400-1), rabbit polyclonal IgG control (300 ng/mouse; BioLegend, CTL-4112), anti-mouse monoclonal IFNAR1 neutralizing antibody (MAR1; 1 μg/mouse; Leinco Technologies, I-401), and mouse IgG control (1 μg/mouse; Leinco Technologies, I-536) were dissolved in sterile PBS and administered intrathecally as described previously.

#### Blocking peptide.

The TAT-KChIP1 blocking peptide (YGRKKRRQRRR-AWLPFARAAAIGWMPVA) was purchased from LifeTein. TAT-KChIP1 was dissolved in sterile saline and administered intrathecally (5 and 10 μg in 5 μL). For control experiments, the TAT-KChIP1 peptide (10 μg) was denatured at 100°C for 30 minutes.

### Behavioral tests

#### Evaluation of thermal sensitivity (hot plate test).

Thermal sensitivity was assessed by the hot plate test (Bioseb). Mice were placed on a metal hot plate set to 52°C ± 0.5°C. The latency from the moment the mouse was placed on the heated surface until it displayed the first overt behavioral sign of nociception, such as lifting or licking a hind paw, vocalization, or jumping, was measured. A timer was used to record the response time, and the mouse was immediately removed from the hot plate after responding or after a maximum cutoff of 30 seconds to prevent tissue damage.

#### Evaluation of thermal hyperalgesia (Hargreaves test).

Thermal hyperalgesia was examined as previously described (21) by measurement of the withdrawal latency of both the right (ipsilateral) and left (contralateral) hind paws using a focused beam of radiant heat (IR = 30) from a Plantar Test apparatus (Ugo Basile). Briefly, mice were individually placed in a small, enclosed testing arena on top of a Plexiglas floor and allowed to acclimate for at least 90 minutes. The Plantar Test apparatus was positioned beneath the animal, so that the radiant heat was directed to the plantar surface of the hind paw. Three trials were performed for each mouse, with a cutoff time set at 15 seconds to prevent tissue damage.

#### Evaluation of mechanical hyperalgesia (von Frey).

Mechanical sensitivity was evaluated using the von Frey test by the method of Dixon (77) adapted by Chaplan et al. (78). Mice were placed individually in small, enclosed testing arenas on top of a wire grid platform and were allowed to acclimate for a period of at least 90 minutes before paw withdrawal threshold (PWT) measurement. Stimulation was applied using the up-and-down method. Calibrated von Frey filaments, in the range 0.02–1.4 g (catalog 58011, Stoelting), were applied perpendicularly to the right hind paw with sufficient force to cause a slight buckling against the paw for 3–5 seconds. A positive response corresponds to a paw withdrawal, flinching, or licking. The PWT was determined as previously described (78).

#### Non-evoked pain behavior measurements.

Non-evoked pain-related behaviors were assessed using the noninvasive animal behavior recognition system LABORAS (Metris). LABORAS is a fully automated behavior recognition and tracking system using mechanical vibrations to classify different natural behaviors (e.g., eating, drinking, climbing, locomotion) and has previously been validated for pharmacological studies (79). Mice were placed in the LABORAS cages for a period of 24 hours, with drinking water and food available ad libitum and under light/dark cycles.

### Mouse DRG neurons

DRG neurons were harvested from mice and enzymatically dissociated in HBSS containing 2 mg/mL collagenase type I and 4 mg/mL dispase (both from Invitrogen) for 45 minutes at 37°C. DRGs were rinsed twice in HBSS and once in Neurobasal A culture medium (Thermo Fisher Scientific) supplemented with 2% B-27, 10% heat-inactivated fetal bovine serum, 100 μg/mL streptomycin, 100 U/mL penicillin, 50 ng/mL nerve growth factor (NGF), and 50 ng/mL glial cell–derived neurotrophic factor (GDNF) (all from Invitrogen). Individual neurons were dispersed by trituration through a fire-polished glass Pasteur pipette in 4 mL medium and cultured overnight at 37°C with 5% CO_2_ in 95% humidity on glass coverslips previously treated with poly-ornithine and laminin (both from Sigma-Aldrich).

### Electrophysiological recordings

DRG neurons were placed in a 1 mL volume chamber and continuously perfused at 1.5 mL/min with extracellular solution containing 140 mM NaCl, 5 mM KCl, 2 mM CaCl_2_, 1 mM MgCl_2_, 10 mM HEPES, and 10 mM d-glucose for current clamp recordings. NaCl was replaced by choline for voltage clamp experiments. Recordings were conducted at room temperature on TdTomato^+^ neurons identified using ×20 magnification on an inverted epifluorescence microscope (IX51, Olympus America Inc.). For the identification of the IB4^+^ neurons, coverslips were treated with the plant lectin IB4 conjugated to fluorescein isothiocyanate (IB4-FITC; 10 μg/mL; Invitrogen, I21411) for 30 minutes. Whole-cell patch clamp experiments were performed in both voltage clamp and current clamp mode using a software-controlled Axopatch 200B amplifier in combination with a Digidata 1550A digitizer. Data were low-pass-filtered at 5 kHz before being sampled at 10 kHz using Clampex 11 (all from Molecular Devices). Borosilicate glass pipettes (Harvard Apparatus Ltd.) were pulled and polished to 2–3 MΩ resistance with a DMZ-Universal Puller (Zeitz-Instruments GmbH). Patch pipettes contained (in mM): K-gluconate 105, KCl 30, MgCl_2_ 4, EGTA 0.3, HEPES 10, Na_2_ATP 4, Na_3_GTP 0.3, and Na-phosphocreatine 10, pH 7.35. All chemicals were purchased from Sigma-Aldrich. Capsaicin was purchased from Sigma-Aldrich (catalog 404-86-4), and AmmTx3 was purchased from Alomone (catalog STA-305).

Under voltage clamp control, cells were held at −70 mV, and membrane capacitance and membrane resistance were calculated in response to a –10 mV hyperpolarizing pulse using the Membrane Test function of Clampex. Resting membrane potential (Vm), rheobase (minimum current injection required to elicit an AP), and resultant AP amplitude of the neurons were evaluated by switching to current clamp mode, prior to returning to voltage clamp control. Access resistance (Ra) and holding current (Ih) were monitored throughout the experiments to ensure recording stability, and recordings with an unstable Ih or poor Ra (initial Ra >20 MΩ or Ra >10% change over the period of recording) were discarded. Data were analyzed from recordings with 75%–95% compensation to minimize offsets due to large voltage clamp errors.

### Data analysis and statistical comparisons

Data were analyzed with Clampfit 11 (Molecular Devices), Easy Electrophysiology (Easy Electrophysiology Ltd.), and Prism 9 (GraphPad).

The peak conductance (*G*) of the potassium current was calculated as follows: *G* = Ip/(Vm – EK), where Ip is the peak outward potassium current, Vm is command voltage, and EK is the estimated reversal potential. Activation and inactivation plots were fitted to the Boltzmann relation: *f*(*V*) = *G*_max_/(1 + exp((*V*_½_ – *V*)/*k*)), where *G*_max_ is the maximal conductance, *V*_½_ is the voltage at which activation or inactivation is half-maximal, and *k* is the slope factor (a positive number for activation, a negative one for inactivation). These parameters were determined from a fit to each experiment, then averaged together to give the mean values (± SEM). To generate averaged, normalized activation and inactivation plots, individual experiments were normalized to *G*_max_ and averaged together. These were refitted to the Boltzmann equation; estimates of *V*_½_ and *k* obtained from these fits differed from the means mentioned above by less than 1 mV.

### Statistics

Statistical analyses were performed with GraphPad Prism 9 software. Normal distribution was verified using D’Agostino-Pearson normality test. For Gaussian data, Student’s 2-tailed *t* test was used to assess statistical significance when comparing 2 means, 1-way ANOVA followed by Tukey’s post hoc test was used to compare more than 2 groups, and 2-way ANOVA followed by Bonferroni’s (2 groups) and Tukey’s post hoc test (for more than 2 groups) was used for multiple comparisons. For non-Gaussian data, the non-parametric Mann-Whitney *U* test was used to assess statistical significance when comparing 2 means, and Kruskal-Wallis followed by Dunn’s post hoc test was used to compare more than 2 groups. Statistical significance was established at *P* ≤ 0.05. Values were expressed as means ± SEM.

### Study approval

Animal studies were conducted according to the protocols approved by the University of Calgary Animal Care Committee (AC19-0169) and the guidelines of the Canadian Council on Animal Care as well as the IACUC at Boston Children’s Hospital and conducted according to institutional animal care and safety guidelines at Boston Children’s Hospital and Harvard Medical School. Human tissues were obtained under the approval of the French institution for organ transplantation (Agence de la Biomédecine, DC-2014-2420) with informed consent from participants and/or their parents/guardians.

### Data availability

All raw data are reported in the Supporting Data Values file. Microarray data from lumbar DRG Nav1.8-Cre Tg-TdTomato neurons from CFA ipsilateral and contralateral sides were deposited at the NCBI’s Gene Expression Omnibus (GEO) database under accession number GSE221834. Microarray data collected from naive lumbar DRG Nav1.8-Cre Tg-TdTomato neurons were previously deposited at the GEO database under accession number GSE46546. Microarray data collected from lumbar DRG TRPV1-ecGFP neurons from CFA ipsilateral and contralateral sides were previously deposited at the GEO database under accession number GSE201227. Values for all data points in graphs have been reported previously (21). Bulk RNA-Seq data collected from lumbar DRG neurons from *GOF* and *TRPV1^cre^-GOF* animals were deposited at the GEO database under accession number GSE236865.

## Author contributions

MD, AB, and CA conceptualized the study. MD, AB, FA, and CA provided experimental design and methodology. MD, AB, FA, NSA, MI, KS, GG, NG, JM, L Bauchet, L Basso, MDC, IMC, VS, AO, GP, NL, FVL, and EB provided investigation. CA provided visualization. CA acquired funding. CA provided project administration. CA provided supervision. MD, AB, IMC, and CA wrote the original draft of the manuscript. MD and CA revised the manuscript. Both co–first authors (MD and AB) played integral roles in addressing technical challenges and uncovering significant findings for the paper. The order among co–first authors, MD and AB, was assigned based on their contributions.

## Supplementary Material

Supplemental data

Unedited blot and gel images

Supporting data values

## Version 1 05/01/2024

Electronic publication
