# Supplementary material for: Induction of antiviral interferon-stimulated genes by neuronal STING promotes the resolution of pain in mice
Source: J Clin Invest. 2024 Mar 19;134(9):e176474. doi: 10.1172/JCI176474 (PMC11060736; doi:10.1172/JCI176474)
Supplement: Supplemental data [file jci-134-176474-s180.pdf]

Induction of antiviral Interferon-Stimulated Genes by neuronal STING promotes the resolution of pain in mice.

This file includes:

Supplemental methods

Supplemental Figure 1 to 12

Supplemental Tables 1 to 5

Supplemental Methods

Type I Interferon measurement

Supernatants of primary DRG neuron cultures were collected 16 hours after stimulation with ADU-S100 (1 µg/ml; Chemietek, CT-ADUS100). To deplete TRPV1 neurons, the primary DRG cultures were treated with Resiniferatoxin (1 µM, RTX, Alomone, R-400) for 15 minutes before ADU-S100 treatment. The concentration of Type I IFN was measured using a Mouse IFN 2-Plex Discovery Assay® (Eve Technologies). Protein concentration was quantified using a Bradford assay (Bio-Rad Laboratories) for normalization.

Microarray analysis of *Nav1.8Tg-TdTomato* Neurons

FACS purification of neurons- 24 hours after CFA injection into the footpad, lumbar (L4-L6) DRGs ipsilateral to or contralateral to the side of CFA injection were dissected from mice, digested in 1 mg/mL Collagenase A/2.4 U/mL Dispase II (enzymes from Roche), dissolved in HEPES buffered saline (Sigma-Aldrich) for 70 minutes at 37°C. For each subsequent microarray sample, DRGs from n=3 mice each were pooled. Following digestion, cells were washed into HBSS containing 0.5% Bovine serum albumin (BSA, Sigma-Aldrich), filtered through a 70 µm strainer, resuspended in HBSS/0.5% BSA, and subjected to flow cytometry. Cells were run through a 100 µm nozzle at low pressure (20 p.s.i.) on a BD FACS Aria II machine (Becton Dickinson, USA). A neural density filter (2.0 setting) was used to allow visualization of large cells and TdTomato+ cells were gated on for isolation. For subsequent RNA extraction, fluorescent neurons were FACS purified directly into 1 mL Qiazol (Qiagen). FACS data was analyzed using FlowJo software (TreeStar, OR USA). Flow cytometry was performed in the IDDRC Stem Cell Core Facility at Boston Children's Hospital.

RNA Processing, Microarray Hybridization and Bioinformatics Analysis-Total RNA was extracted by sequential Qiazol extraction and purification through the RNeasy micro kit with on column genomic DNA digestion according to the manufacturer's instructions (Qiagen, CA, USA). RNA quality was determined by Agilent 2100 Bioanalyzer using the RNA Pico Chip (Agilent, CA, USA). Samples with RIN>7, were used for subsequent analysis. RNA was amplified into cDNA using the Ambion WT expression kit for Whole Transcript Expression Arrays, with Poly-A controls from the Affymetrix GeneChip Eukaryotic Poly-A RNA control kit (Affymetrix, CA, USA). The Affymetrix GeneChip WT Terminal labeling kit was used for fragmentation and biotin labeling. Affymetrix GeneChip Hybridization control kit and the Affymetrix GeneChip Hybridization, wash, stain kit was used to hybridize samples to Affymetrix Mouse Gene ST 1.0 GeneChips, fluidics performed on the Affymetrix GeneChip Fluidics

Station 450, and scanned using Affymetrix GeneChip Scanner 7G (Affymetrix). Microarray work was conducted at the Boston Children's Hospital IDDRC Molecular Genetics Core facility. Affymetrix CEL files were normalized using the Robust Multi-array Average (RMA) algorithm with quantile normalization, background correction, and median scaling. Differentially expressed transcripts were illustrated using volcano plots, generated by plotting fold-change differences against comparison p-values or –log (p-values).

Microarray analysis of *TRPV1-pHluorin* Neurons

Flow Cytometry-On day 3 of CFA, both ipsilateral and contralateral DRG sample (L4-L6) from TRPV1-ecGFP mice were collected and digested separately. After digestion, cells were filtered through a 90 mm mesh (Sarstedt) and washed in PBS 1% FBS. Cells were analyzed on a FACS Aria II (BD Bioscience). After FACS sorting, RNA was extracted separately from GFP positive and GFP negative cells using a RNeasy Mini kit (Qiagen). The quantity of RNA was determined using a Nanodrop 2000c spectrophotometer (Thermo-Fisher Scientific).

RNA Processing, Microarray Hybridization and Bioinformatics Analysis-Three biological replicates for the GFP positive and GFP negative samples (~50 ng/sample) were submitted to the Centre for Applied Genomics (Toronto, Canada). Here the quality of the samples was assessed using the Agilent Bioanalyzer 2100 with the RNA Pico chip kit (Agilent Technologies). RNA integrity number values between 6.5 and 7 were achieved. The expression profiling was performed according to the manufacturer's instructions with Affymetrix GeneChip Mouse Gene 2.0 ST Array (Affymetrix). Primary data analysis was carried out with the Affymetrix Expression Console 1.4.1.46 software including the RMA module for normalization. Gene expression data were log-transformed. A change was considered significant when the FDR-corrected p-value/q-value thresholds met the criterion $q < 0.01$ at fold changes > 2 (expression increments or declines larger than two).

RNA sequencing analysis of *TRPV1*^{cre}*-GOF* Neurons

RNA Processing and sequencing

Neurons-Lumbar (L4-L6) DRGs were dissected from naïve *GOF* (n= 4) and *TRPV1*^{cre}*-GOF* mice (n= 4). Total RNA from was purified through the RNeasy micro kit with on column genomic DNA digestion according to the manufacturer's instructions (Qiagen, CA, USA). RNA quality was determined by Agilent Technologies 5200 Fragment Analyzer (Agilent, CA, USA). Samples with RIN>7, were used for

subsequent analysis. cDNA libraries were generated were generated using the NEBNext® Ultra™ II Directional RNA Library Prep Kit for Illumina® (New England Biolabs) on an Illumina NextSeq500.

RNA sequencing analysis

The quality control of reads was performed with FastQC, and all samples passed quality control. Reads were then aligned with STAR (v2.4.0j) (80) on the Mus musculus genome GRCm39.107 from EBI databases. Alignment was then converted with SAMtools (v1.16.1) (81), and genes counts were computed with HTSeq counts (v0.9.1) (82).

Differential Gene Expression analysis - Count matrix from HTSeq-counts were analyzed with DESeq2 (v1.34.0) (83). In order to compare samples, raw data were normalized with rlogTransformation function. A change was considered significant when the adjusted p-value (Benjamini-Hochberg) thresholds met the criterion $p<0.05$ at fold changes > 2 (expression increments or declines larger than two).

Analysis of published single-cell RNAseq

To analyze STING (*Tmem173*) expression in peripheral sensory neurons, the «Adolescent, Level 6, Taxonomy Level 2 Pns neurons» dataset from the mouse brain atlas (http://mousebrain.org/) (22) was extracted and processed using Seurat package (Seurat version 4.1.0) (59) in R (version 4.1.1). Loom files were converted into Seurat object using the LoadLoom function from SeuratDisk package. Results were then processed with the classical Seurat pipeline. The expression matrix for each population was computed with AverageExpression function, and the heatmap was generated using the DoHeatmap function of the mean expression matrix of the list of gene of interest.

Immunohistochemistry

DRGs and Spinal cord - Animals were perfused with phosphate buffered saline (PBS) to wash out blood and then perfused with 4% paraformaldehyde (PFA) (Electron Microscopy Science, Cat. No. 15713). Lumbar (L4-L6) DRGs and spinal cord were extracted and dehydrated overnight in 30% sucrose and embedded in optimal cutting temperature (OCT) solution (VWR International, Cat. No. 95057-838). Embedded tissues were sliced 10-µm thick onto SuperFrost slides (VWR International). The following antibodies were used in this study: chicken polyclonal anti-GFP (1:500, Invitrogen, A10262), rabbit polyclonal anti-GFP (1:500, Chromotek, # PABG1), rabbit polyclonal anti-STING (1:100, Cell Signaling, 13647S), rabbit polyclonal anti-TRPV1 (1:250, Alomone, ACC-030), guinea pig polyclonal anti-TRPV1 (1:250, Alomone, ACC-030-GP), rat polyclonal anti-Substance P (1:500, Millipore, MAB356), IB4-

coupled Alexa 594 (1:1000, Invitrogen, I21412), IB4-coupled Alexa 488 (1:1000, Invitrogen, I21411), goat polyclonal anti-GFRα3 (1:500, R&D Systems,VFU021721), goat polyclonal anti-GFRα2 (1:500, R&D Systems, AF429), donkey anti-chicken Alexa Fluor 488 (1:1000, Invitrogen, SAB4600031), goat anti-rabbit Alexa Fluor 488 (1:2000, Invitrogen, A11008), goat anti-rabbit Alexa Fluor 555 (1:2000, Invitrogen, A21428), chicken anti-goat Alexa Fluor 647 (1:2000, Invitrogen, Cat. No. A21469;), anti-rat Alexa Fluor 647 (1:2000, Cederlane, 712-605-153).

All sections were mounted using Aqua PolyMount (Polysciences). Confocal images were acquired on a Zeiss LSM 510 Meta confocal microscope and AxioCam HRm camera and analyzed using a 20× objective. Sections were imaged and analyzed using ImageJ. A minimum of three mice per group were analyzed.

DRG neurons culture - DRG cultures treated with Resiniferatoxin were fixed for 15min with PFA 4%. To characterize the depletion of TRPV1 neurons, coverslips were incubated for 1-hour at room temperature with chicken polyclonal anti-GFP (1:500, Invitrogen, A10262) and a rabbit polyclonal anti-β-tubulin III (1:1000, Sigma-Aldrich, # T2200). Afterward, coverslips were washed in PBS, incubated with secondary antibody mix (donkey anti-chicken Alexa Fluor 488 (1:1000, Invitrogen, SAB4600031) and goat anti-rabbit Alexa Fluor 555 (1:2000, Invitrogen, A21428)) and mounted using Aqua PolyMount (Polysciences). Confocal images were acquired on a Zeiss LSM 510 Meta confocal microscope and AxioCam HRm camera and analyzed using a 10X objective. Sections were imaged and analyzed using ImageJ.

In situ hybridization

Human DRG neurons- RNAscope in situ hybridization multiplex chromogenic assay was performed as instructed by Advanced Cell Diagnostics (ACD). Snap frozen human DRG tissues were cryo-sectioned at 18 µm, mounted on SuperFrost Plus slides (Fisher Scientific) and stored at -80°C. The next day, slides were removed from the -80°C freezer and immediately washed with PBS (pH 7.4; 5 min, twice), fixed with 4% PFA-PBS, and then dehydrated in 50% ethanol (5 min), 70% ethanol (5 min) and 100% ethanol (5 min) at room temperature. Slides were pre-treated with H2O2 10 min at RT and washed twice in distilled water. Then, slides were submerged in 1X boiling RNAscope target retrieval reagent for 5 minutes. After target retrieval agent treatment, slides were transferred in distilled water and then washed in 100% ethanol. The slides were air dried briefly and then boundaries were drawn around each section using a hydrophobic pen (ImmEdge PAP pen; Vector Labs). When hydrophobic boundaries have dried, protease III reagent was added to each section and slides were incubated for 20 min. at 40°C in a HybEZ

oven (ACD). This last step was repeated once, and slides were washed with distilled water before RNAscope assay. The RNAscope assay was performed according to the manufacturer's instructions using a HybEZ oven (ACD). The probes used were *Hs-TMEM173* (ACD, # 433541), *Hs-SCN10A* (ACD, #406291-C2). At the end of the process, hematoxylin counterstain was performed (30% Gill hematoxylin n°1; Sigma-Aldrich) and slides were mounted with Vectamount mounting medium (Vector Labs). Slides were imaged at 40x with Hamamastu Nanozoomer 2 slide scanner.

Mouse DRG neurons- Co-detection of RNAscope in situ hybridization multiplex v2 fluorescent assay (ACD, #323110) combined with immunofluorescence was performed on mouse DRG neurons. Mice were euthanized with isoflurane and then perfused with PBS followed by 4% PFA as described above. DRGs were extracted and placed in 4% PFA overnight at 4°C followed by 30% sucrose overnight at 4°C and then embedded in OCT and stored at -80°C. OCT sections were cut into 10μm slices onto SuperFrost slides and stored at -80°C for up to two months. On the day of the assay, slides were washed In PBS (5 min, twice, RT), baked for 30 min at 60°C and then post-fixed in pre-chilled 4% PFA in PBS for 15 min at 4°C. The tissue was then dehydrated sequentially in 50% ethanol (5 min, RT), 70% ethanol (5 min, RT), and 100% ethanol (2 min, twice, RT). The tissue was then treated with H2O2 (10 min, RT) (ACD, #322335), washed twice in PBS with 0.1% Tween-20 (PBS-T) (Sigma-Aldrich, #P1379) (5 min, twice, RT), and then a barrier was created around the tissue using a hydrophobic pen (Vector Labs, #H-4000). The primary antibody, diluted in Co-detection Antibody Diluent (ACD, #323160) was applied to each section and stored overnight at 4°C. The following day, the tissue was washed in PBS-T (2 min, twice, RT), and then submerged in 10% Neutral Buffered Formalin (NBF) (30 min, RT) for post-primary fixation. The tissue was then washed in PBS-T (2 min, 4 times, RT), RNAscope Protease Plus (ACD, #322331) was applied to the tissue for 30 min at 40°C. The RNAscope assay was then performed according to ACD's instructions. At the end of the assay, before mounting the slides, the secondary antibody was added to the tissue for 30 min at RT before the slide was washed in PBS-T (2 min, twice, RT). The slides were then mounted using ProLong Gold antifade reagent with DAPI (Invitrogen, #P36931). The probes used were *Mm-Trpv1* (ACD, #313331-C2), *Mm-Kcnip1* (ACD, #466891-C3), *Hs-TMEM173* (ACD, #433541), and *Mm-Ifnar1* (ACD, #512971) and were labelled using TSA Vivid Fluorophores 570 (ACD, #323272) and 650 (ACD, #323273). The primary antibody used was anti-GFP (Chromotek, #PABG1) and the secondary antibody was Alexa Fluor 488 donkey anti-rabbit IgG (Invitrogen, #A21206). The tissue was imaged using the Leica SP8 confocal microscope at 20x or 63x objectives.

Western Blot Analysis

TBK1 expression-To look at the phosphorylation of TBK1, we performed primary DRG neurons culture from WT and STING-/- mice and stimulated them with 10 µg/ml or 30 µg/ml ADU-S100 for one, three or six hours. DRGs were homogenized using a bullet blender (Next Advance) with SSB02 beads (Next Advance) and lysed in RIPA buffer with protease and phosphatase inhibitors (Thermo-Fisher Scientific) for 45 minutes. Lysates were centrifuged at 10,000g for 10 min at 4°C, supernatants were collected, and protein concentration was quantified and normalized using a Bradford assay (Bio-Rad laboratories). Total lysates were separated by SDS-PAGE and transferred onto nitrocellulose membranes (Sigma-Aldrich). Membranes were blocked in 5% BSA 1h at room temperature, and then probed with anti-phospo-TBK1 antibody (1:200 dilution in 1% BSA, Cell Signalling, 5483S) at 4°C overnight. Membranes were then washed three times with TBS-T and incubated with horseradish peroxidase (HRP)-conjugated anti-rabbit antibodies (1:1000 in 1% BSA; Cederlane, NA934) for 1h at room temperature. Bands were visualized using the Immobilon Western chemiluminescent HRP Substrate (Bio-Rad Laboratories), and band density was calculated using Image J. Intensity of anti-TBK1 antibody (1:1000 dilution in 1% BSA; Cell signalling, 3504S) band was used for normalization among samples.

KChIP1 expression- DRGs were homogenized using a bullet blender (Next Advance) with SSB02 beads (Next Advance) and lysed in RIPA buffer with protease and phosphatase inhibitors (Thermo-Fisher Scientific) for 45 minutes. Lysates were centrifuged at 10,000g for 10 min at 4°C, supernatants were collected, and protein concentration was quantified and normalized using a Bradford assay (Bio-Rad laboratories). Total lysates were separated by SDS-PAGE and transferred onto nitrocellulose membranes (Sigma-Aldrich). To investigate the level of KChIP1 protein, membranes were blocked in 5% BSA 1h at room temperature, and then probed with anti-KChIP1 antibody (1:250 dilution in 1% BSA, Alomone, APC-141.) at 4°C overnight. Membranes were then washed three times with TBS-T and incubated with horseradish peroxidase (HRP)-conjugated anti-rabbit antibodies (1:1000 in 1% BSA, Cederlane, NA934) for 1h at room temperature. Bands were visualized using the Immobilon Western chemiluminescent HRP Substrate (Bio-Rad Laboratories), and band density was calculated using Image J. Intensity of Rabbit anti-Beta-tubulin III antibody (1:1000 dilution in 1% BSA; Sigma-Aldrich, T2200) band was used for normalization among samples.

Cytokine profiling

Blood samples were centrifuged (5000 x g, 30 minutes, 4°C) and then serum was processed using both a Mouse IFN 2-Plex Discovery Assay® and a MILLIPLEX Mouse Cytokine Array Proinflammatory Focused 10-plex (Eve Technologies).

qPCR

DRGs and paws were harvested and dissociated using a bullet blender (Next Advance) with SSB02 beads (Next Advance) in RLT buffer (Qiagen). Total RNA was extracted using a RNeasy Mini kit (Qiagen), according to the manufacturer's instructions. The quality and quantity of RNA were determined using a Nanodrop 2000c spectrophotometer (Thermo-Fisher Scientific). Relative gene expression (normalized to GAPDH) was determined by qPCR using BlasTaq 2X qPCR MasterMix (ABMgood, G892) and a StepOnePlus real-time PCR detection system (Applied Biosystems). The designed primers for DNA amplification are listed in Supplemental **Supplemental Table 5**.

Supplemental Figures

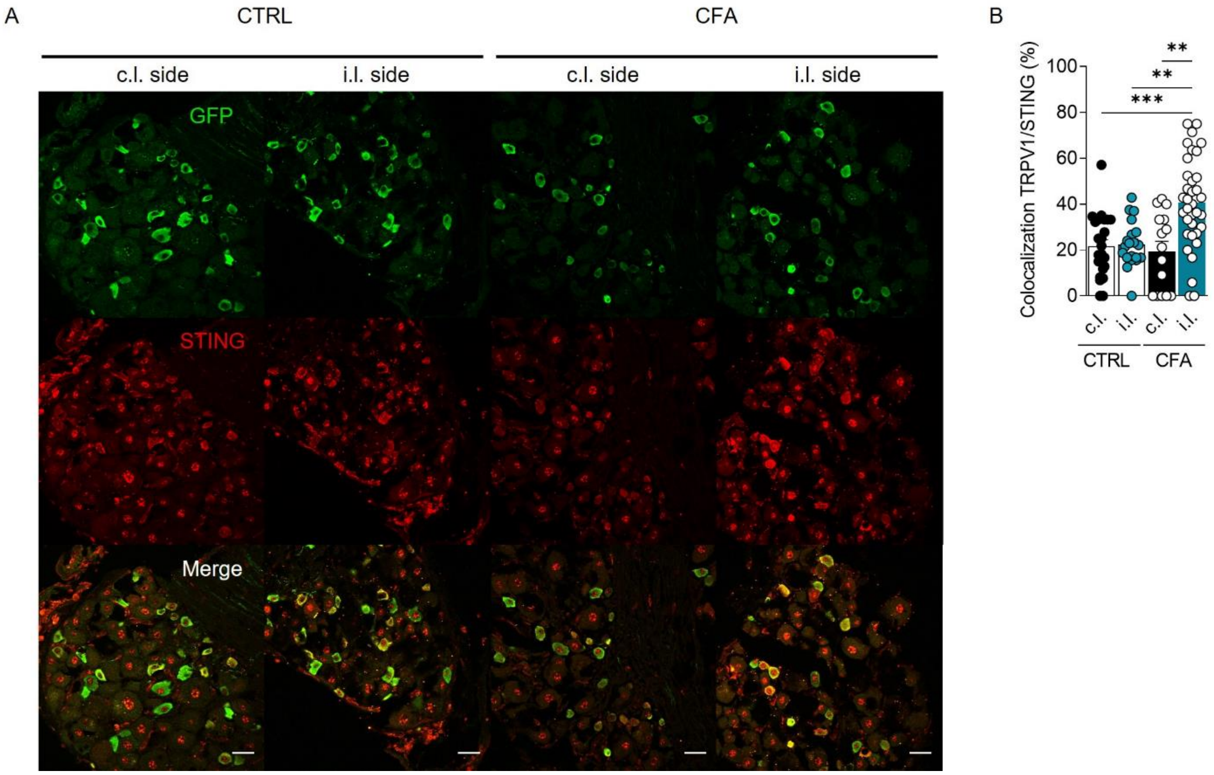

Supplemental Figure 1: Upregulation of STING protein in TRPV1 neurons during CFA. (A) Representative confocal images of co-immunostaining for STING and TRPV1-ecGFP in both ipsilateral and contralateral DRG neurons of CFA-treated mice. Scale bars: 50 µm. (**B**) Dot plot showing the percentage of neurons co-expressing STING and TRPV1 in DRG from *TRPV1-ecGFP* mice (each symbol represents a single DRG section; CTRL c.l. n = 24; CTRL i.l. n = 20; CFA c.l. n = 15; CFA i.l. n = 35 from 3 mice). Statistical analysis was performed using Kruskal-Wallis followed by Dunn’s post-hoc test (** $p<0.01$, *** $p<0.001$).

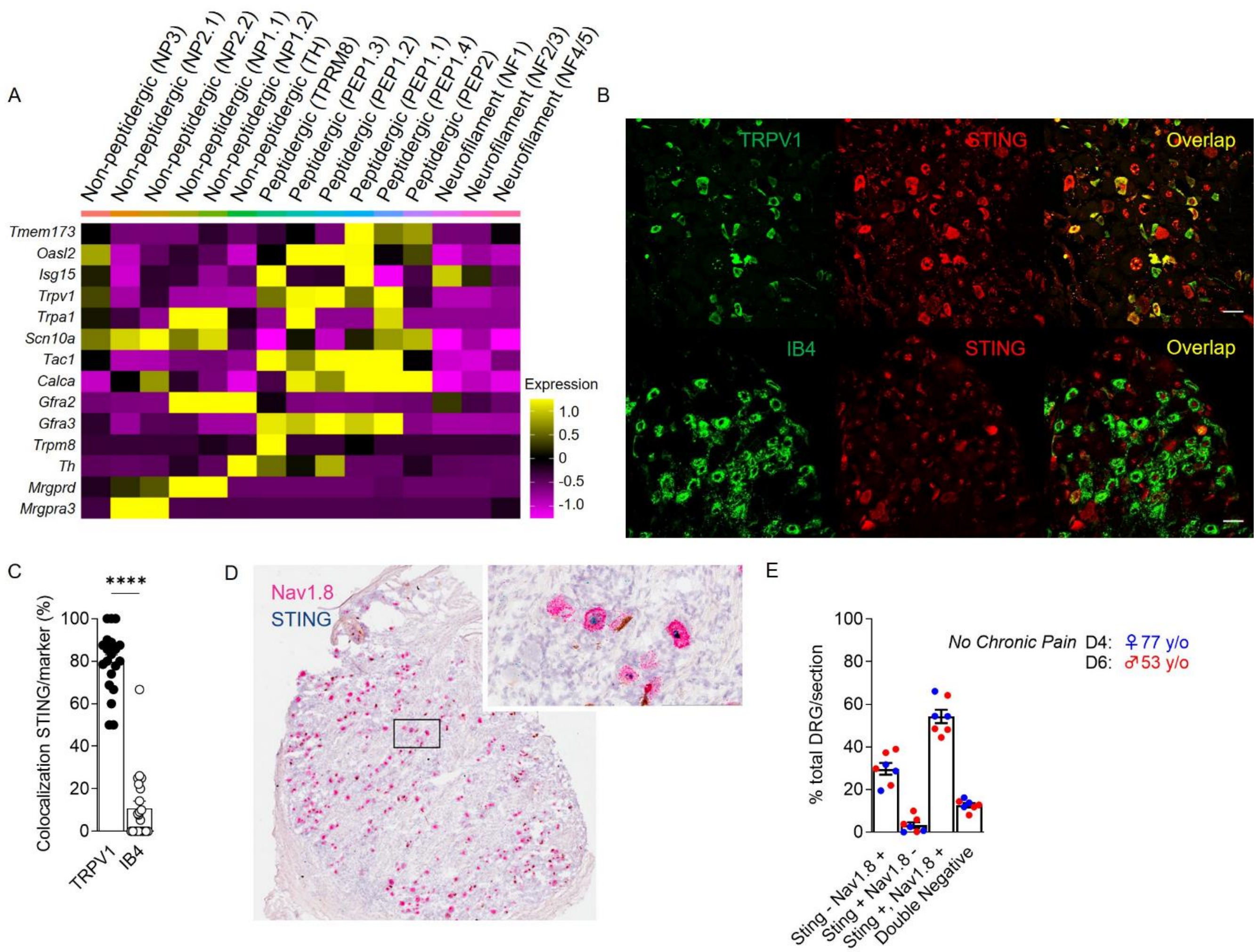

Supplemental Figure 2. STING is expressed in mouse and human DRG nociceptors. (A) Heatmap of the expression of *Sting* and selected population markers on the 15 populations of sensory neurons from DRG described in Zeisel et al., (22). (**B**) Representative confocal images of co-immunostaining for STING, IB4 and TRPV1 in mouse DRG neurons. Scale bars: 50 µm. (**C**) Dot plot showing the percentage of peptidergic (TRPV1) and non-peptidergic (IB4) neurons co-expressing STING in DRG from TRPV1-pHluorin mice (each symbol represents a single DRG section; TRPV1+, n = 22; IB4+, n = 20 from 3 mice). Statistical analysis was performed using t-test (**** p<0.001). (**D**) Representative RNAscope image showing expression of *STING* (light blue) in human DRG neurons co-expressing *SCN10A* (Nav1.8) (pink). (**E**) The bar graph summarizes the results (each symbol represents a single DRG; n = 3 for one individual and n = 4 for another individual).

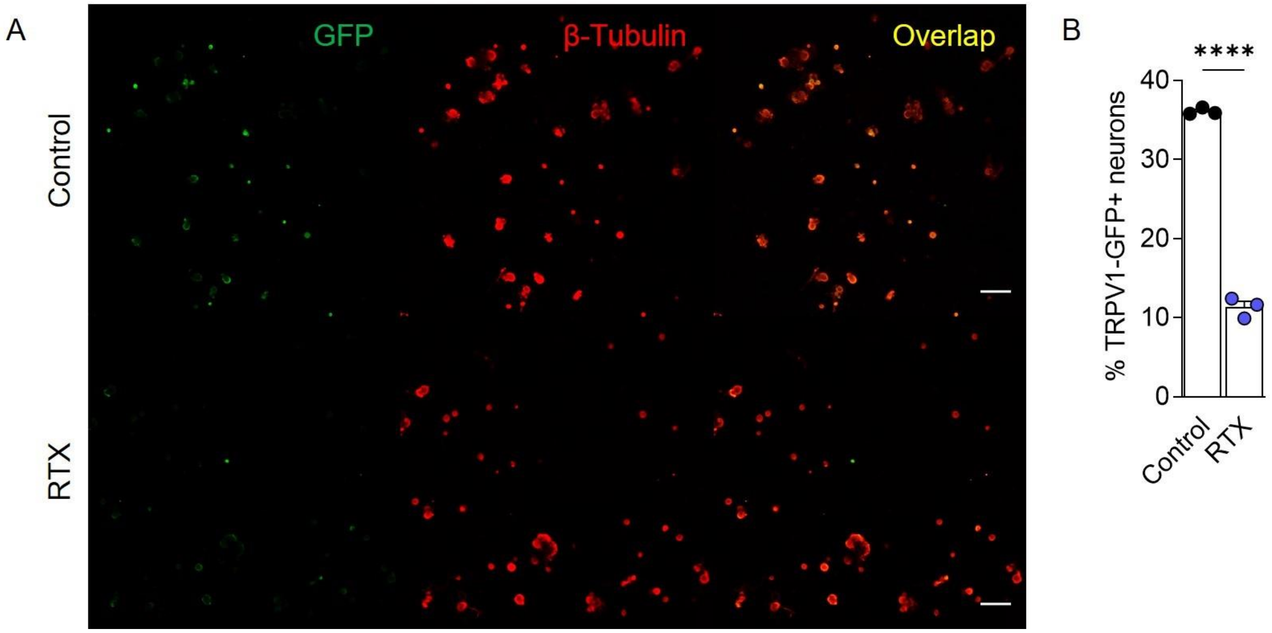

Supplemental Figure 3. Pharmacological depletion of TRPV1 neurons. (A) Representative confocal images of co-immunostaining for TRPV1-GFP and β-Tubulin in primary culture of mouse DRG neurons treated with RTX (1 µM) or vehicle. Scale bars: 50 µm. (**B**) Dot plot showing the percentage of TRPV1-GFP positive neurons treated with Vehicle (n = 3 mice) or RTX (n = 3 mice). Statistical analysis was performed using T-test (**** p<0.001).

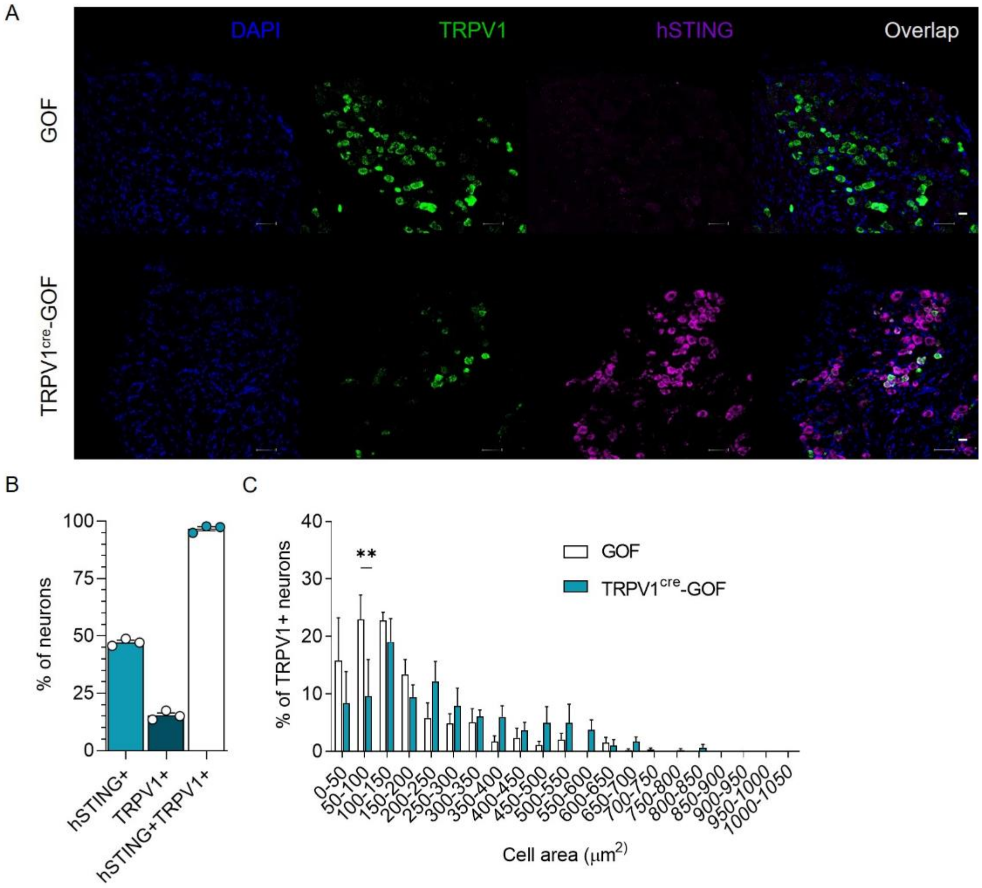

Supplemental Figure 4. **Nociceptor-specific *hSTING-N154S* mutation alters the numbers of TRPV1 neurons**. (**A**) Expression of *hSTING* and TRPV1 in DRGs from *TRPV1^{Cre}-GOF* and *GOF* littermate controls. Scale bars: 50 µm. (**B**) The bar graph summarizes the percentage of hSTING+ and TRPV1+ neurons normalized to the total number of neurons. Note that ~100% of hSTING+ neurons are TRPV1+ neurons (each symbol represents a single mouse, n = 3). (**C**) Size distribution of the percentage of DRG neurons immunopositive for TRPV1, in naïve *GOF* (n = 3) and *TRPV1^{cre}-GOF* mice (n = 3). Statistical analysis was performed using Two-way ANOVA followed by Bonferroni's post-hoc test (** p<0.01).

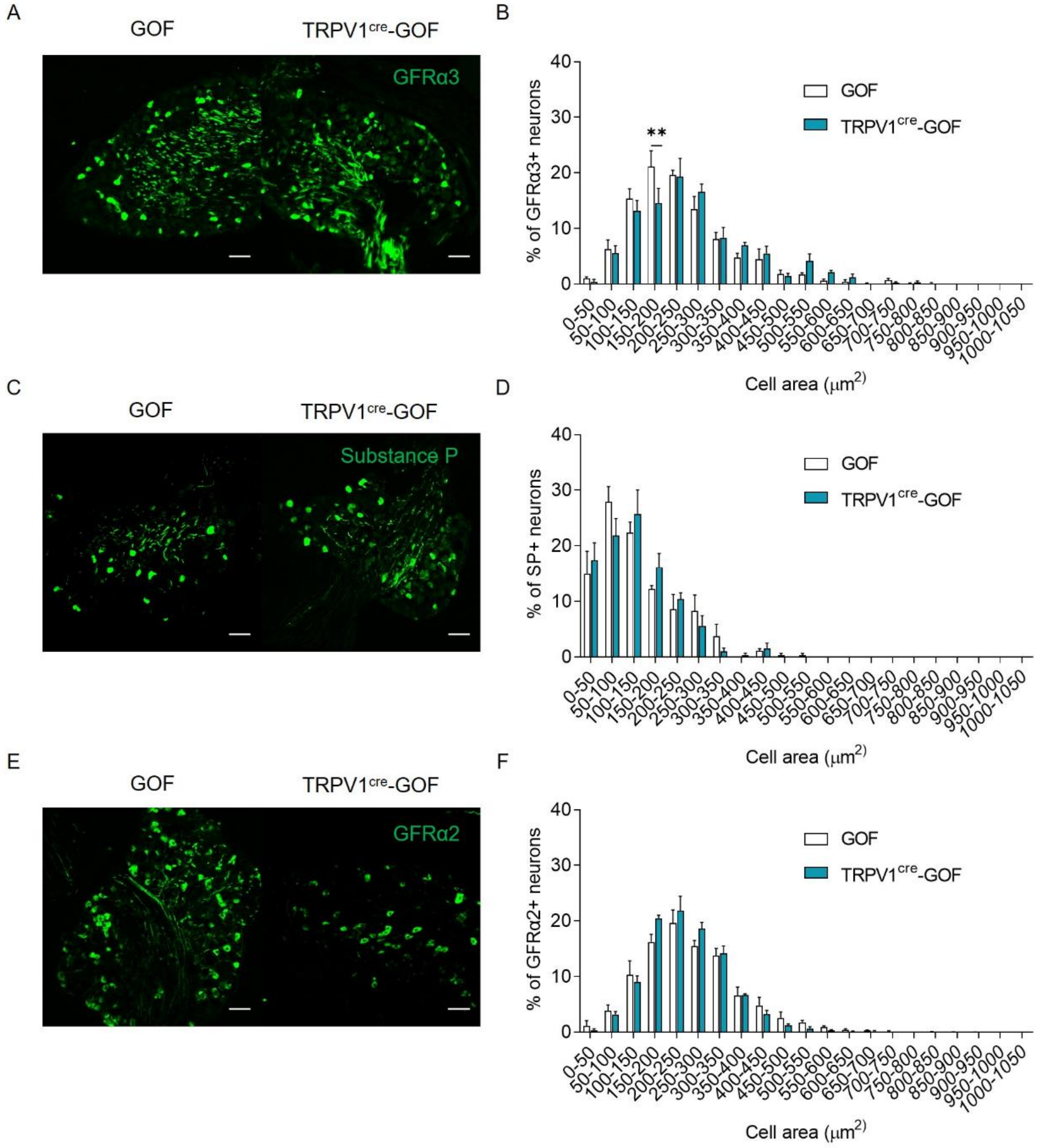

Supplemental Figure 5. **Characterization of peptidergic and non-peptidergic neurons in mice expressing the nociceptor-specific *hSTING-N154S* gain of function mutation.** Representative confocal images of immunostaining for GFRα3 (**A**), Substance P (**C**) and GFRα2 (**E**) in DRGs of *TRPV1^{Cre}-GOF* and *GOF* mice. Scale bars: 50 µm. Size distribution of DRG neurons positive for GFRα3 (**B**), Substance P (**D**) and GFRα2 (**F**) in *TRPV1^{Cre}-GOF* (n = 3-4) and *GOF* mice (n = 4-5). Statistical analysis was performed using Two-way ANOVA followed by Bonferroni's post-hoc test (** $p<0.01$).

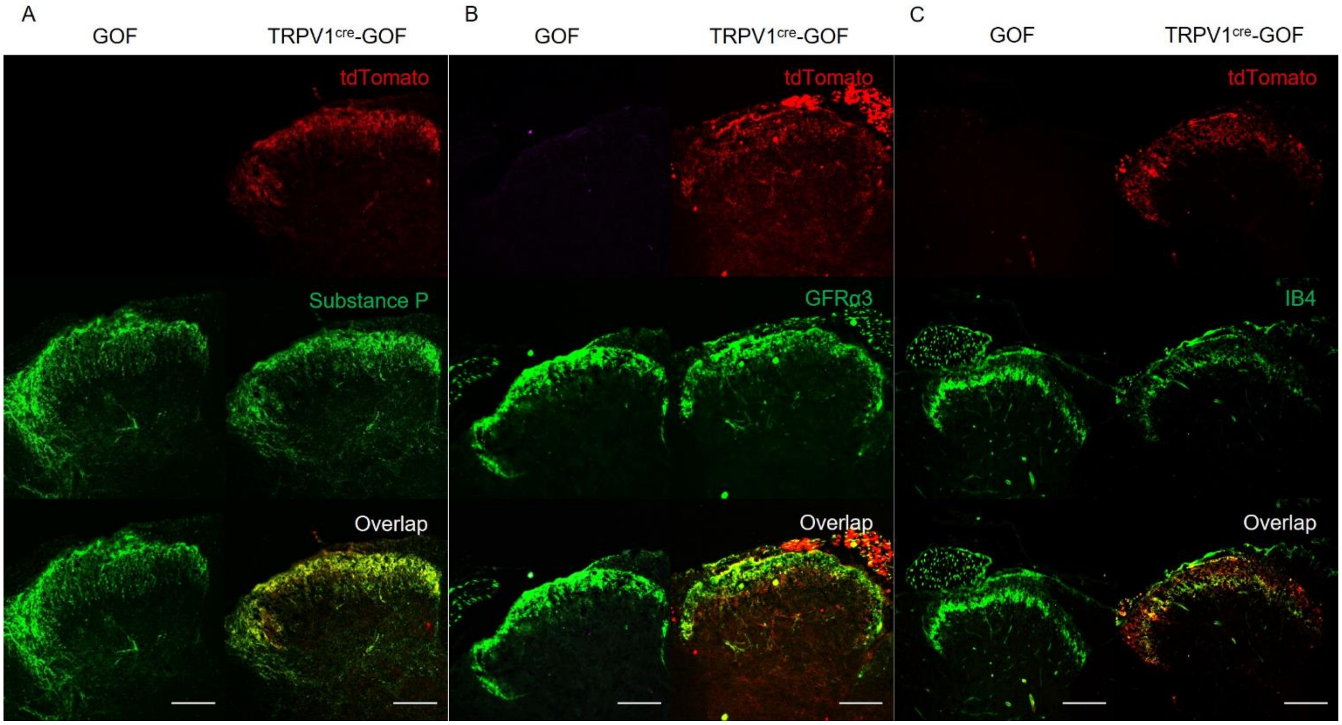

Supplemental Figure 6. **Nociceptor-specific *hSTING-N154S* mutation does not alter the neuronanatomical organization of TRPV1 neurons**. Representative confocal images of lumbar spinal cord section from *TRPV1^{Cre}-GOF* and *GOF* littermate controls, immunostained for Substance P (**A**), GFRα3 (**B**) and IB4 (**C**). Scale bars: 100 µm.

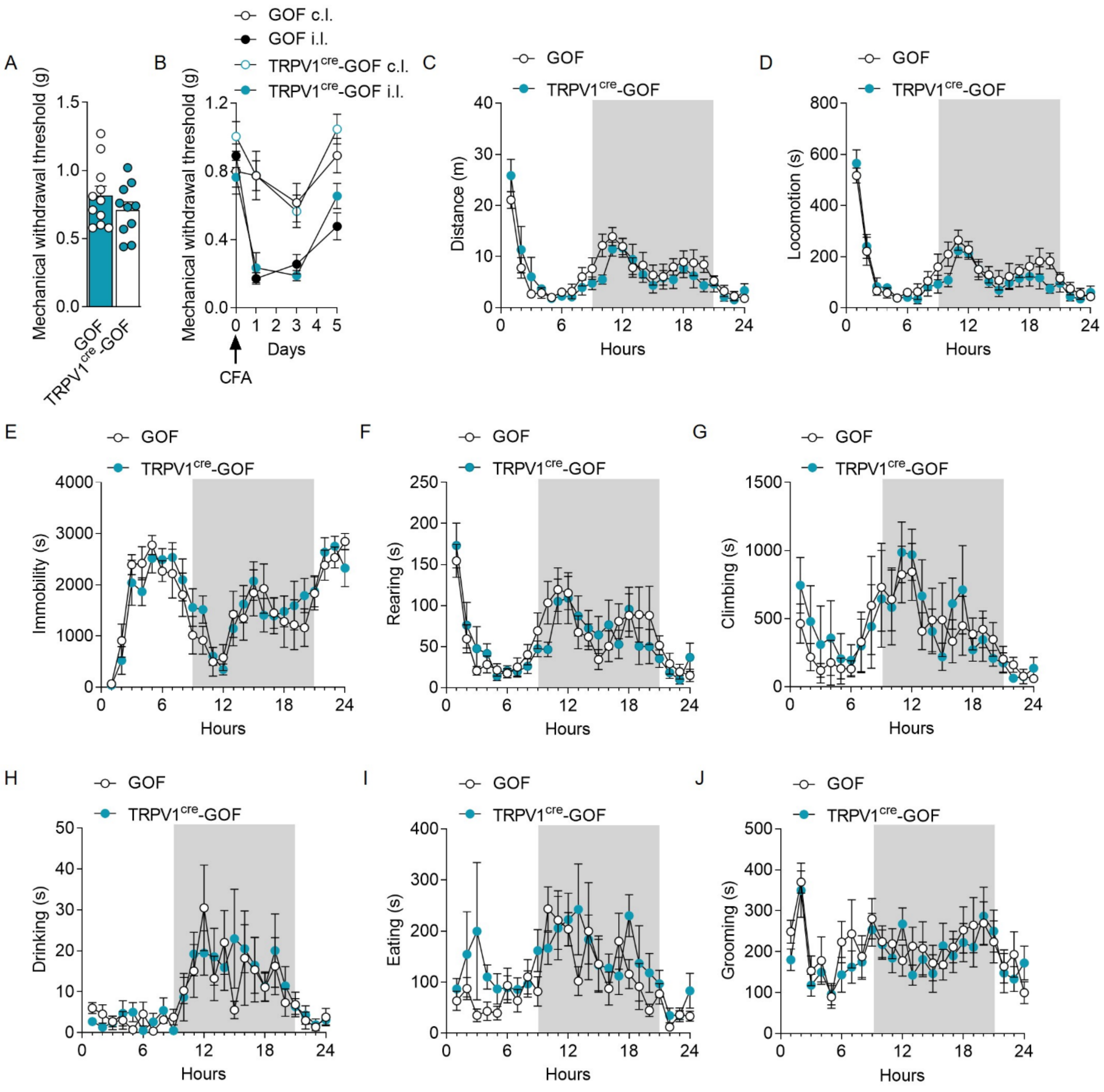

Supplemental Figure 7. **Mice expressing the nociceptor-specific *hSTING-N154S* mutation exhibit no changes in non-evoked pain-related behaviors and mechanical hyperalgesia. (A)** Mechanical sensitivity in naïve *TRPV1^{Cre}-GOF* and *GOF* littermate controls (n=10 and 11, respectively). **(B)** Measurement of mechanical allodynia in *TRPV1^{Cre}-GOF* and *GOF* littermate controls following intraplantar CFA (n = 6 and 8, respectively). **(C-J)** Non-evoked normal behaviors of both naïve *GOF* (n = 8) and *TRPV1^{cre}-GOF* (n = 9) mice. Distance **(C)**, Locomotion **(D)**, Immobility **(E)**, Rearing **(F)**, Climbing **(G)**, Drinking **(H)**, Eating **(I)**, Grooming **(J)** behaviors were measured using the Laboras system for 24 hours. (**A**) Statistical analysis was performed using a t-test. (**B-J**) Statistical analysis was performed using Two-way ANOVA followed by Tukey post-hoc test (**B**) or by Bonferroni's post-hoc test (**C-J**).

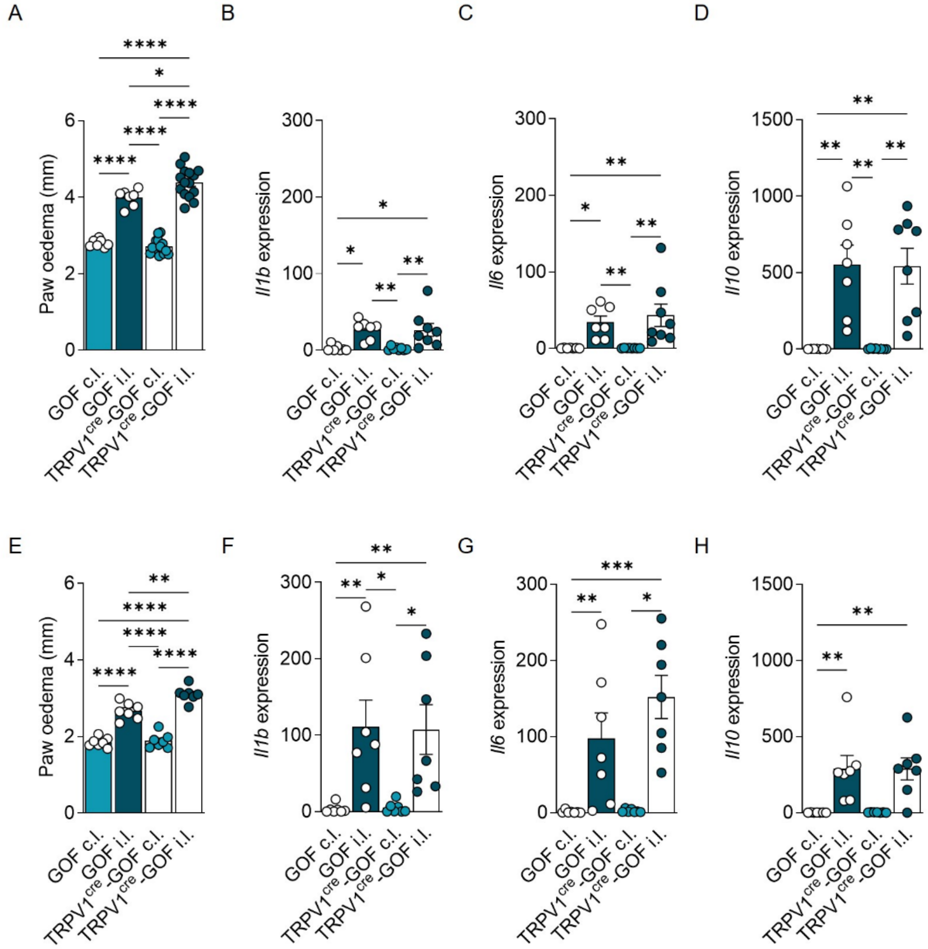

Supplemental Figure 8: **Nociceptor-specific *hSTING-N154S* mutation does not alter the inflammatory response in the paw** (**A**) Measurement of oedema in the ipsilateral and contralateral hind paws at day 3 of CFA-treated *GOF* mice (n = 7) and *TRPV1^cre^-GOF* mice (n = 15). *Il1b* (IL-1) (**B**), *Il6* (IL-6) (**C**) and *Il10* (IL-10) (**D**) mRNA were determined by RT-qPCR at day 3 of CFA-treated *GOF* (n = 7), and CFA-treated *TRPV1^cre^-GOF* (n = 8) mice. (**E**) Measurement of edema in the ipsilateral and contralateral hind paws at day 12 of CFA-treated GOF mice (n = 7) and TRPV1^{cre}-GOF mice (n = 7). *Il1b* (IL-1) (**F**), *Il6* (IL-6) (**G**) and *Il10* (IL-10) (**H**) mRNA were determined by RT-qPCR at day 12 of CFA-treated *GOF* (n = 7), and CFA-treated *TRPV1^{cre}-GOF* (n = 7) mice. Statistical analysis was performed using One-Way ANOVA followed by Tukey post-hoc test (**A-B;E**) (* p<0.05, ** p<0.01, **** p<0.0001) or Kruskal-Wallis followed by Dunn's post-hoc test (**C-D;F-H**) (* p<0.05, ** p<0.01, *** p<0.001).

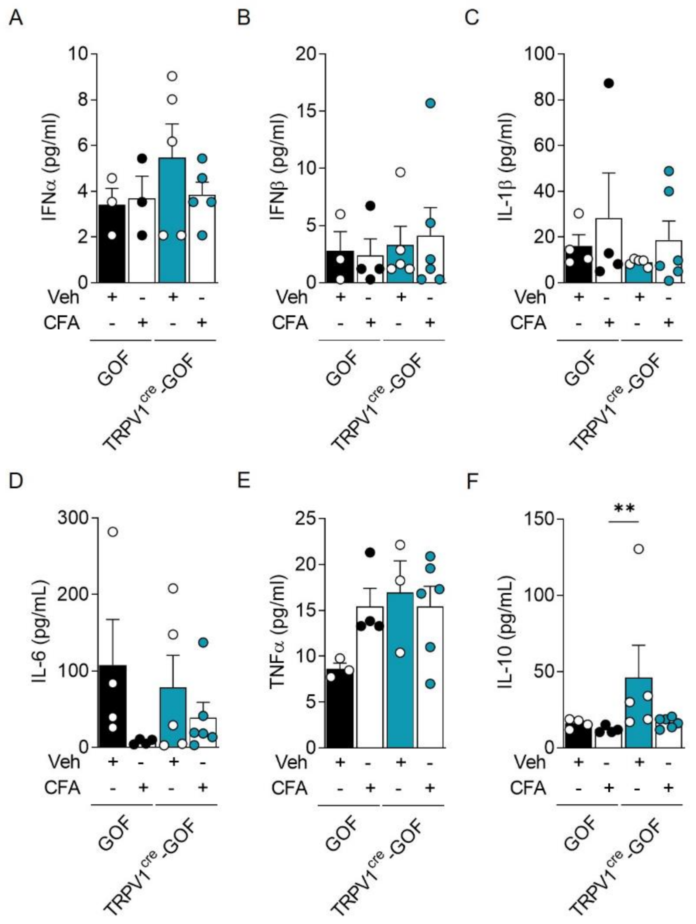

Supplemental Figure 9. **Nociceptor-specific *hSTING-N154S* mutation does not induce systemic inflammation.** IFNα (**A**), IFNβ (**B**), IL-1β (**C**), IL-6 (**D**), TNF-α (**E**) and IL-10 (**F**) levels were determined by Luminex technology in the serum of *GOF* (n = 3-4), *TRPV1^{cre}-GOF* (n = 3-5), CFA-treated *GOF* (n = 3-4) and CFA-treated *TRPV1^{cre}-GOF* (n = 5-6) mice. Statistical analysis was performed using One-Way ANOVA by Tukey post-hoc test (**A**) or Kruskal-Wallis followed by Dunn's post-hoc test (**B-F**).

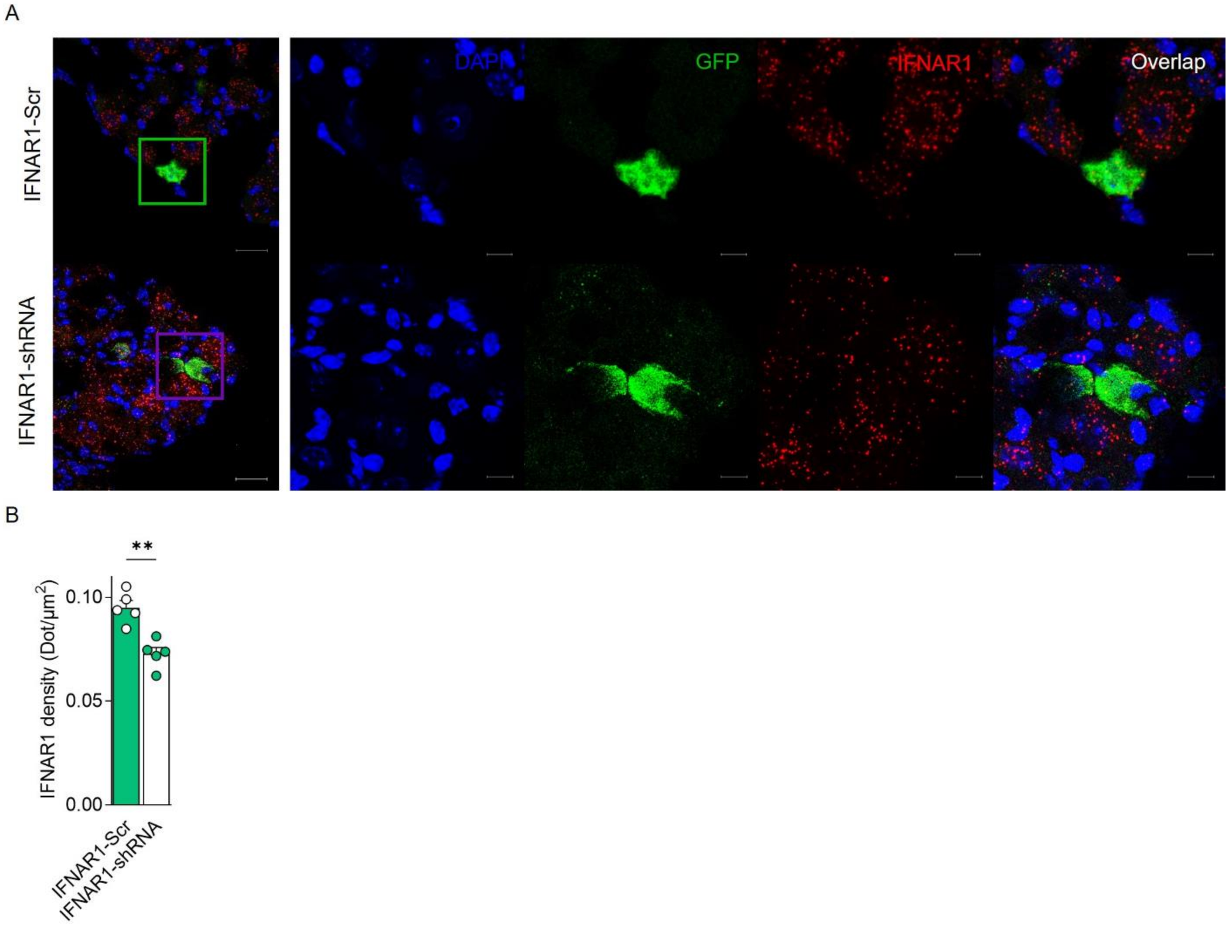

Supplemental Figure 10. **Knockdown of *Ifnar1* in TRPV1 neurons from *TRPV1^{Cre}-GOF* mice. (A)** Representative confocal image of TRPV1 neurons from *TRPV1^{Cre}-GOF* mice injected with *AAV-DIO-Scr-shRNA* (n = 5) or *AAV-DIO-IFNAR1-shRNA* (n=5). Images represent DAPI staining, AAV-GFP expression (green) and *Ifnar1* transcripts (red) by RNAscope. Scale bars: 25 µm and 10 µm on the cropped images. **(B)** Quantification of *Ifnar1* density measured by the number of transcripts represented by dots per surface unit in AAV-infected TRPV1 neurons. Statistical analysis was performed using unpaired t-test (** $p<0.01$).

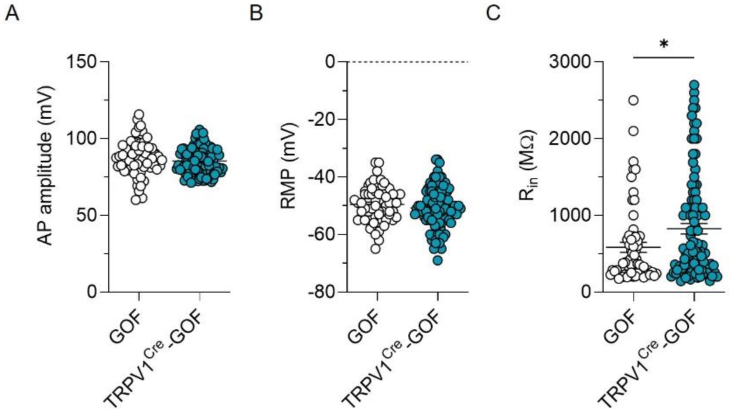

Supplemental Figure 11. Electrophysiological properties of *TRPV1-hSTING-N154S* expressing neurons. (A) Measurement of action potential amplitude in TRPV1 neurons from *TRPV1^{Cre}-GOF* mice and *GOF* littermate controls. **(B)** Measurement of the resting membrane potential of TRPV1 neurons from *TRPV1^{Cre}-GOF* mice and *GOF* littermate controls. **(C)** Measurement of the input resistance of TRPV1 neurons from *TRPV1^{Cre}-GOF* mice and *GOF* littermate controls (n = 61 for *GOF* and n = 101 for *TRPV1^{Cre}-GOF*). Statistical analysis was performed using unpaired t-test (* $p<0.05$).

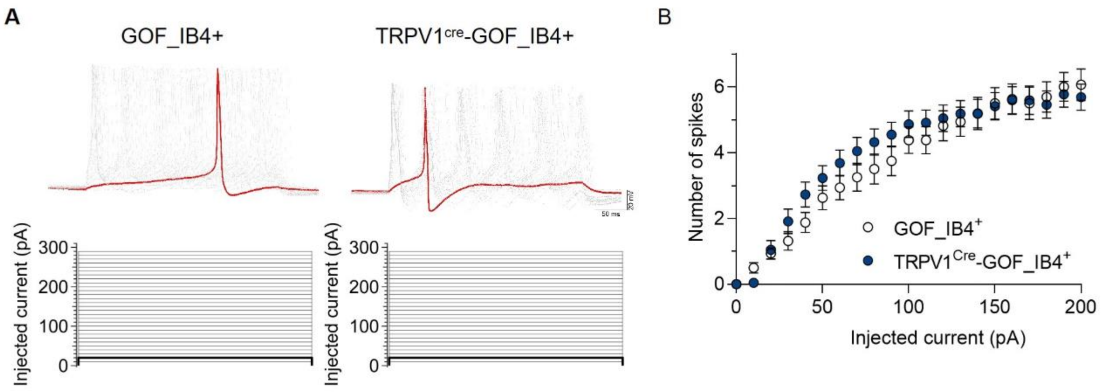

Supplemental Figure 12. Nociceptor-specific *STING-N154S* gain of function mutation does not alter the excitability of non-peptidergic neurons. (A) Representative current clamp recording of IB4+ neurons from *TRPV1^{Cre}-GOF* mice and *GOF* littermate controls (top panel). Cells were injected with 500 ms current pulses with an increment of 10 pA and an interval of 5 sec (protocol, bottom panel). The highlighted black line indicates the current amplitude that induces the first action potential. Scale bars: 20mV/50ms. **(B)** Number of spikes as a function of injected current in IB4+ neurons from *TRPV1^{cre}-GOF* mice and *GOF* littermate controls. Statistical analysis was performed using a two-way ANOVA test with Tukey's post-hoc test.

Supplemental Tables

Supplemental Table 1. Differentially expressed genes. Upregulated and downregulated genes with statistical significance and fold change between ipsilateral (CFA) and contralateral groups. Significant genes were selected by fold change (>2.0 or < 0.5-fold) and adjusted p-value (<0.05).

**Upregulated**				**Downregulated**			
Affymetrix ID	Genes	Fold Change	p-value	Affymetrix ID	Genes	Fold Change	p-value
10471555	*Angptl2*	2.426091091	0.001208263	10508719	*Snora16*a	0.452314335	0.021619405
10458314	*Tmem173*	2.298822724	0.008794532	10342630		0.497983723	0.048657737
10485261	*Accsl*	2.167894708	0.016933286				
10598976	*Timp1*	2.839586997	0.02308973				

Supplemental Table 2. Cytokine profiling was determined by Luminex technology in the serum of GOF (n=4), TRPV1^{cre}-GOF (n=5), CFA-treated GOF (n=4) and CFA-treated TRPV1^{cre}-GOF (n=6) mice. Statistical analysis was performed using Kruskal-Wallis followed by Dunn's post-hoc test.

Cytokine	GOF Control (pg/ml ± SEM)	GOF CFA (pg/ml ± SEM)	TRPV1^{cre}-GOF Control (pg/ml ± SEM)	TRPV1^{cre}-GOF CFA (pg/ml ± SEM)	P value
GM-CSF	34.70 ± 3.228	26.16 ± 1.229	32.96 ± 2.764	28.81 ± 2.745	0.1114
IL-2	20.78 ± 4.683	11.17 ± 1.127	10.19 ± 1.093	13.04 ± 1.072	0.0596.
IL-4	4.415 ± 1.244	1.500 ± 0.4823	3.448 ± 1.815	1.457 ± 0.2974	0.1088
IL-12	95.55 ± 20.62	39.28 ± 6.904	239.6 ± 172.8	43.86 ± 6.527	0.1292
MCP-1	334.3 ± 190	136.3 ± 15.44	322.2 ± 115.6	121.1 ± 11.25	0.2296
IFN-y	N.D.	N.D.	N.D.	N.D.	N.D.

Supplemental Table 3. Differentially expressed genes. Upregulated and downregulated genes with statistical significance and fold change between TRPV1^{cre-}GOF and GOF mice. Significant genes were selected by fold change (>1.0 or < –1.0-fold) and adjusted p-value (<0.05).

**Upregulated**			**Downregulated**		
Genes	log2FoldChange	padj	Genes	log2FoldChange	padj
XR_882529.2	7.261004098	6.48E-05	*Mdfic2*	-1.072121203	1.02E-06
XR_387206.3	6.58321701	8.24E-05	*Ly86*	-1.122940057	0.000323776
Apol9a	5.759672042	2.97E-05	*Prkcq*	-1.254490097	0.000602701
NM_023124.5	5.648729299	0.003055314	*Rasgrp1*	-1.279973961	6.48E-05
C130026I21Rik	5.597484827	0.008376753	*Trpv1*	-1.322091607	0.000244676
Ccl5	5.427265998	1.64E-12	*Gm7271*	-1.422580965	5.05E-07
Ifit1	5.418747143	4.31E-119	*Rgs8*	-1.621563051	0.035381852
Serpina3m	5.37739573	0.016609153	*Ccdc68*	-1.676407073	0.004020513
Apol9b	5.234133712	0.00948795	*A3galt2*	-1.739081513	0.027137132
Gm41228	5.002376966	0.001515469	*XR_001779761.1*	-2.473259254	0.021160254
Zbp1	4.721015141	3.36E-10	*Ms4a3*	-2.803452221	0.001488239
Isg15	4.693520896	1.98E-103	*C1ql4*	-3.060333376	0.006405778
AA465934	4.632499794	5.44E-08	*Sst*	-3.744073782	8.00E-11
XR_385292.2	4.4654571	0.000580895			
XM_006530316.3	4.400342531	1.17E-55			
XM_006518531.2	4.342546244	0.003358037			
Phf11b	4.333980896	3.28E-18			
Oasl2	4.184997956	6.60E-68			
Ifi44	4.019245493	0.000477605			
Ifih1	3.924828644	4.17E-20			
Oasl1	3.842998029	2.00E-05			
Usp18	3.77470373	7.10E-16			
H2-Q6	3.71768066	5.92E-10			
Cxcl10	3.68980293	1.40E-14			
XM_006526706.3	3.662732381	4.33E-07			

H2-Q9	3.612317692	0.035782145
Rtp4	3.607941282	3.79E-23
Ifi27l2a	3.601126487	8.34E-80
XM_011247152.2	3.502554196	0.04041286
Mx1	3.441815263	0.012994557
Dhx58	3.373440082	2.82E-06
H2-Q5	3.275821344	3.24E-06
Rsad2	3.270226059	2.41E-13
XM_006502553.2	3.245476311	0.001342011
Ifit3	3.22816284	3.99E-34
Oas1a	3.215921251	1.66E-08
Irf7	3.141628278	1.26E-19
Herc6	3.118816927	0.000351353
H2bc12	2.901872421	0.013019598
Gbp3	2.893669424	6.78E-15
Pcdh8	2.856204653	0.025364631
Sp110	2.803035033	0.000628121
Sp100	2.744130554	0.004278155
Oas1b	2.706692439	0.026844712
Cd274	2.677055418	9.67E-06
Hdc	2.663072117	0.035381852
Dnase1l3	2.557386704	4.14E-07
Gbp6	2.498480634	0.009051305
Ifit3b	2.485087282	9.63E-10
Ddx58	2.446117645	1.62E-09
Tgtp2	2.436337437	0.007681859
Mctp2	2.387737983	3.06E-07
XM_006501721.2	2.34140457	0.022722137
XM_006523701.2	2.338889506	3.98E-05
Isg20	2.329620597	5.50E-06
Gbp2	2.279056055	1.05E-11
Bst2	2.233539497	4.89E-19

Gbp7	2.227655494	3.73E-06
Psmb8	2.21296197	5.89E-20
B2m	2.148447769	1.41E-74
Psmb9	2.140728516	3.42E-07
H2-Q4	2.131235809	4.02E-25
Gbp5	2.123585345	0.018346107
Xaf1	2.118456599	3.58E-14
Cpox	2.051738348	2.22E-14
Ifi47	2.041813917	0.010206924
H2-K1	2.036665596	3.85E-62
XM_006532305.2	2.004585755	0.000477605
Samd9l	1.98004085	0.000127774
Stat2	1.938177014	6.95E-10
Tap1	1.899903981	0.001342011
Zc3hav1	1.897077762	0.036746434
Trim12c	1.895575702	0.003086056
Gdf11	1.858508336	0.000126789
Igtp	1.841257986	4.88E-09
Casp1	1.774448922	0.005267098
Parp10	1.773058804	0.009376392
Parp14	1.745782138	0.049270562
H2-T23	1.737961793	2.65E-13
Cnr1	1.736883741	0.034464934
XM_006523680.2	1.719485177	0.006173653
H2-D1	1.694018875	1.23E-58
Psmb10	1.629375891	1.93E-13
C4b	1.615511146	9.27E-07
Kcnip1	1.583650624	1.94E-07
Lgals9	1.563781076	1.72E-20
Tmem140	1.559678147	0.044860189
Lgals3bp	1.517871062	3.51E-12
Stat1	1.495573463	0.003593545

Cmpk2	1.494597383	2.54E-05
Chl1	1.447212398	0.013019598
XM_006495811.3	1.366995836	0.025026029
Ly6e	1.350298134	8.29E-14
H2-T22	1.341226133	0.034987023
Irf9	1.309244363	0.002710968
Ifitm3	1.273454262	2.48E-20
Mrgprx1	1.213282434	0.006946101
XM_006509970.3	1.192701653	0.000945973
Slc23a2	1.174372544	0.023152055
Irf1	1.154626843	0.011803336
Pcp4	1.05673494	1.14E-20
Ifi35	1.043831809	0.004020513
Fxyd3	1.035158707	0.002021108
Psme1	1.022284316	5.50E-06
Ube2l6	1.003625237	0.001677066

Supplemental Table 4. Primer sequences Genotyping

Genotype	**Sequence 5' --> 3'**	**Primer Type**
Flox-hSTING-N154S	ATACCTTTCTGGGAGTTCTCTGCTG	Forward
	CACACACCAGGTTAGCCTTTAAGC	Reverse
	GGGCGTACTTGGCATATGATACAC	Reverse
TRPV1-cre	GCG GTC TGG CAG TAA AAA CTA TC	Mutant Forward
	GTG AAA CAG CAT TGC TGT CAC TT	Mutant Reverse
	TTC AGG GAG AAA CTG GAA GAA	Wild type Forward
	TAG TCC CAG CCA TCC AAA AG	Wild type Reverse

Supplemental Table 5. qPCR primer sequences

Gene	Sequence 5' --> 3'
Oasl2	TTGTGCGGAGGATCAGGTACT
	TGATGGTGTCGCAGTCTTTGA
Isg15	CAATGGCCTGGGACCTAAA
	CTTCTTCAGTTCTGACACCGTCAT
Trpv1	AACAAGAAGGGGCTTACACC
	TCTGGAGAATGTAGGCCAAGAC
Kchip1	CGACCCTCCAAAGATAAGATTG
	AGTTCCTCTCAGCAAAATCGAC
Ifnar1	GCAGTGTGACCTTTTCAGCA
	GAGAATTCACACTTGGTCGTTG
Gapdh	ATGCTGGTGCTGAGTATGTCG
	GTGGTGCAGGATGCATTGCTGA
IL1b	ACCTTCCAGGATGAGGACATGAG
	CATCCCATGAGTCACAGAGGATG
Il6	TCTGGGAAATCGTGGAAATGAG
	TTCTGCAAGTGCATCATCGTTG
Il10	ATTTGAATTCCCTGGGTGAGAAG
	CACAGGGGAGAAATCGATGACA
