# Supplementary material for: Induction of antiviral interferon-stimulated genes by neuronal STING promotes the resolution of pain in mice
Source: J Clin Invest. 2024 Mar 19;134(9):e176474. doi: 10.1172/JCI176474 (PMC11060736; doi:10.1172/JCI176474)
Supplement: Unedited blot and gel images [file jci-134-176474-s181.pptx]

## Slide 1
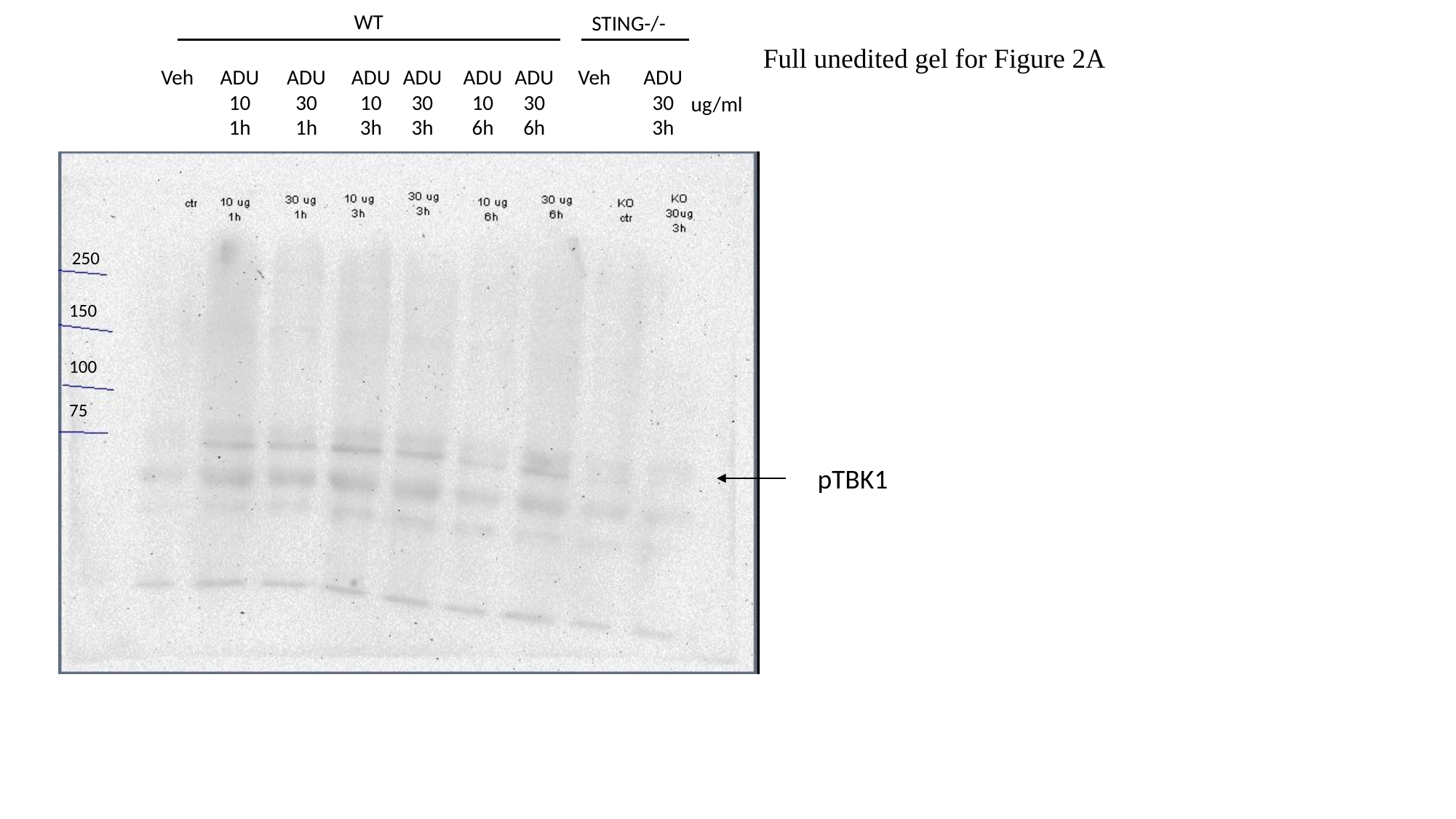

WT
STING-/-
Full unedited gel for Figure 2A
Veh
ADU 10 1h
ADU 30 1h
ADU 10 3h
ADU 30
3h
ADU 10
6h
ADU 30 6h
Veh
ADU 30 3h
ug/ml
250
150
100
75
pTBK1

## Slide 2
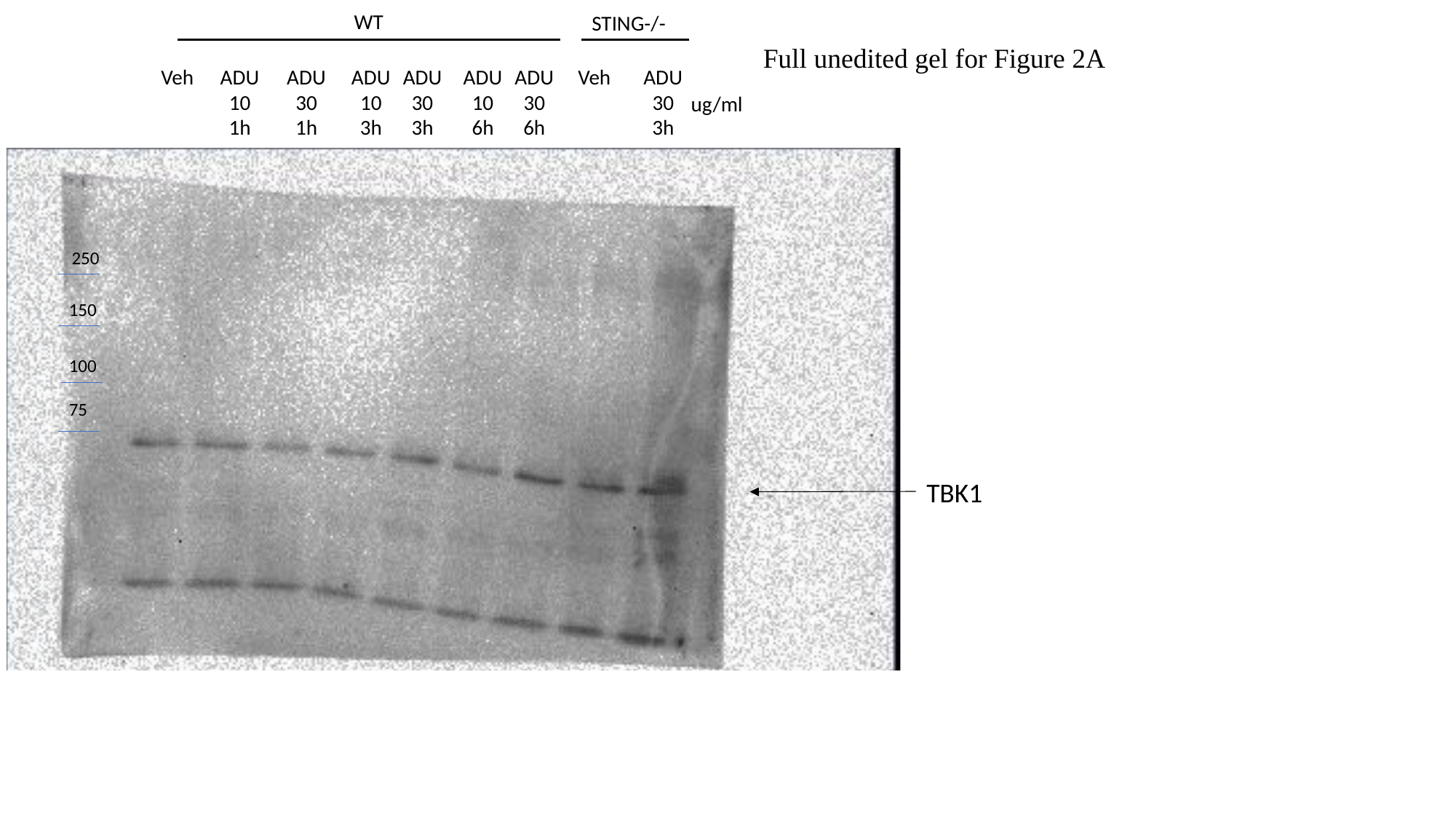

WT
STING-/-
Full unedited gel for Figure 2A
Veh
ADU 10 1h
ADU 30 1h
ADU 10 3h
ADU 30
3h
ADU 10
6h
ADU 30 6h
Veh
ADU 30 3h
ug/ml
250
150
100
75
TBK1

## Slide 3
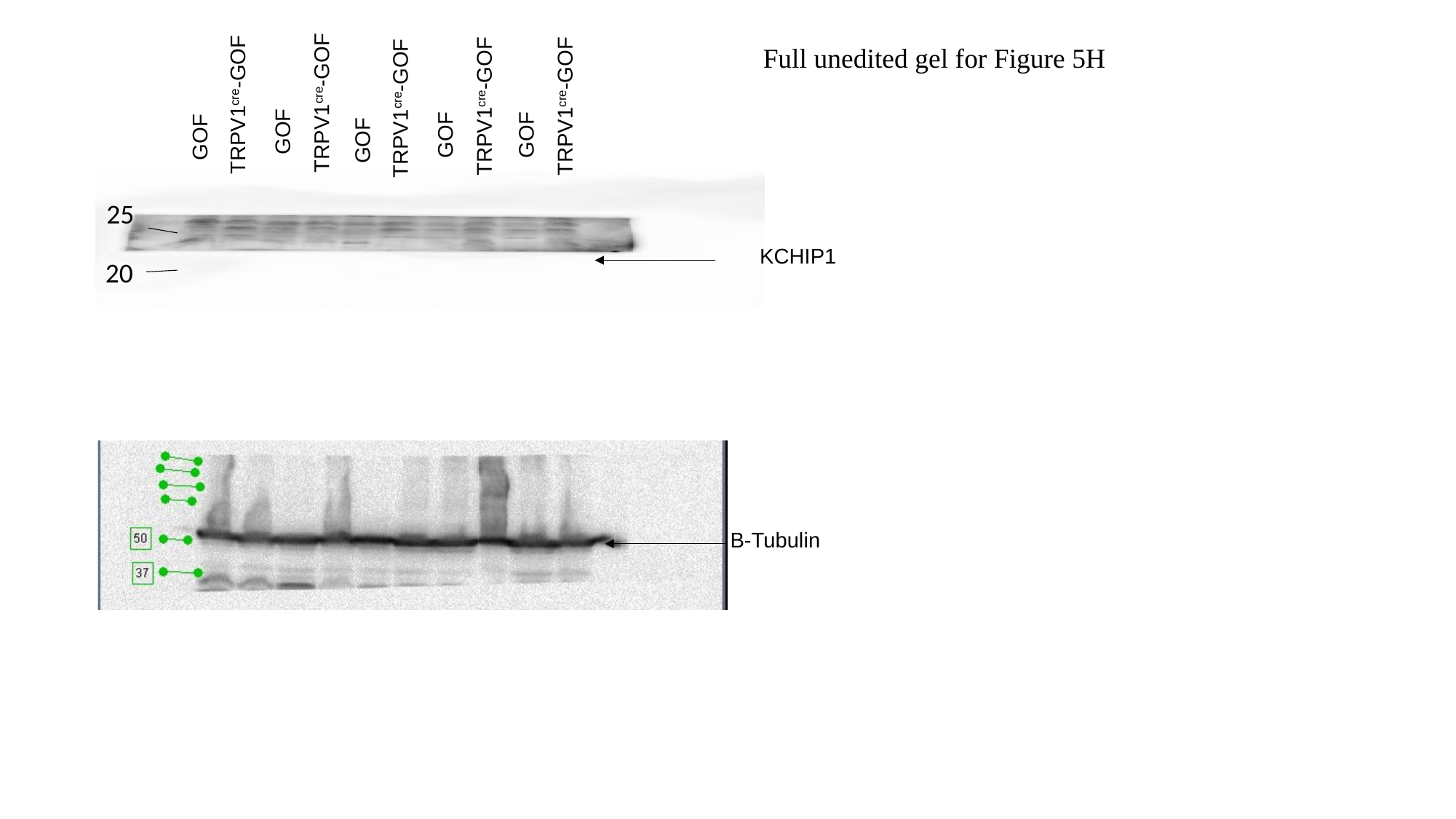

Full unedited gel for Figure 5H
GOF
GOF
GOF
GOF
GOF
TRPV1cre-GOF
TRPV1cre-GOF
TRPV1cre-GOF
TRPV1cre-GOF
TRPV1cre-GOF
25
KCHIP1
20
B-Tubulin
